# New Bicyclic Cembranoids from the South China Sea Soft Coral *Sarcophyton trocheliophorum*

**DOI:** 10.1038/srep46584

**Published:** 2017-04-21

**Authors:** Lin-Fu Liang, Wen-Ting Chen, Xu-Wen Li, He-Yao Wang, Yue-Wei Guo

**Affiliations:** 1 State Key Laboratory of Drug Research, Shanghai Institute of Materia Medica, Chinese Academy of Sciences, Shanghai 201203, China; 2 College of Material Science and Engineering, Central South University of Forestry and Technology, Changsha 410004, China

## Abstract

Nine new bicyclic cembranoids, sarcophytrols M–U(1–9), were isolated from the South China Sea soft coral *Sarcophyton trocheliophorum* as minor components, along with one known related cembranoid 10. Their structures were elucidated by detailed spectroscopic analysis and chemical conversion. The chemical structures of these metabolites are characterized by the different patterns of the additional cyclization within the 14-member skeleton, which leading to the formation of furan, pyran, oxepane, and peroxyl rings, respectively. Among them, sarcophytrols R and S(6 and 7) share a rare decaryiol skeleton with an unusual C12/C15 cyclization. In addition, the absolute configurations of sarcophytrols M and T(1 and 8) were determined by the modified Mosher’s method. The research of these new secondary metabolites provided a further understanding of the diversity of cyclized cembranoids from the title species.

Cyclization is an extraordinary artistry that nature turn the simple cembranoids to a prodigious variety of structurally novel compounds, and it often links to a network of oxygenation process^1^, which lead to the formation of epoxyl^2^,^3^,^4^,^5^, furan^4^,^6^,^7^,^8^, pyran^5^,^7^,^8^, and oxepane^5^,^9^,^10^. Among them, the decaryiol-type cembranoids, characterized by a 6:14-fused ring system, are one of the most amazing examples. They are biogenetically derived from cembranoids by an uncommon transannular etherfication between C-12 and C-15 position and rarely discovered in nature. Actually, there are only four decaryiol-type cembranoids, decaryiols A–D, that have been reported from soft coral *Sarcophyton decaryi*^11^ and *Lobophytum* sp^12^. before.

It is widely recognized that the South China Sea soft coral *S. trocheliophorum*(phylum Cnidaria, class Anthozoa, subclass Octocorallia, order Alcyonacea, family Alcyoniidae) contains unusual cembranoids with a diversity of cyclizations^13^. Interestingly, these cembranoids are regarded as chemical defense compounds against predators such as other corals and fishes as well as against settlements of microorganisms^14^,^15^. In our ongoing studies of the chemistry and biology of the Hainan soft corals, we have considered the soft coral *S. trocheliophorum* as an important issue. A previous study we conducted on a collection of the title animal from Hainan had resulted in the isolation of a series of new cembranoids and cembranoid derivatives^3^,^4^,^9^,^16^. Many of these new secondary metabolites with different patterns of cyclizations showed significant inhibitory activity against human protein tyrosine phosphatase 1B(PTP1B) enzyme^3^,^4^,^16^, a promising drug target for the treatment of type 2 diabetes and obesity^17^. To accumulate these compounds for further biological study, we made a different collection of the same species from the same location(Yalong Bay, Hainan Province). Surprisingly, our chemical investigation on the crude acetone extract of the title animal showed the absence of aforementioned compounds existed in the former collection, while resulting to the discovery of three new capnosane diterpenoids^18^. We have now focused our attentions on the cembrane-type metabolites with diverse kinds of cyclizations from the latter collection to find more chemically interesting and biologically active compounds. This continuous investigation has now resulted in the isolation of nine new cembranoids(**1**–**9**), together with one known related cembranoid **10**(Fig. 1). Among them, the characteristic chemical features of them are the diverse types of cyclized rings: furan rings possessed by sarcophytrols M–P(**1**–**4**), pyran rings formed at different positions in sarcophytrols Q–S(**5**–**7**), while oxepane and peroxyl rings appeared in sarcophytrols T(**8**) and U(**9**), respectively. In addition, sarcophytrols R and S(**6** and **7**) share a rare decaryiol skeleton with an unusual C12/C15 cyclization. We herein report the isolation and structure elucidation of these new cembranoids.

## Results and Discussion

**Figure 1:**
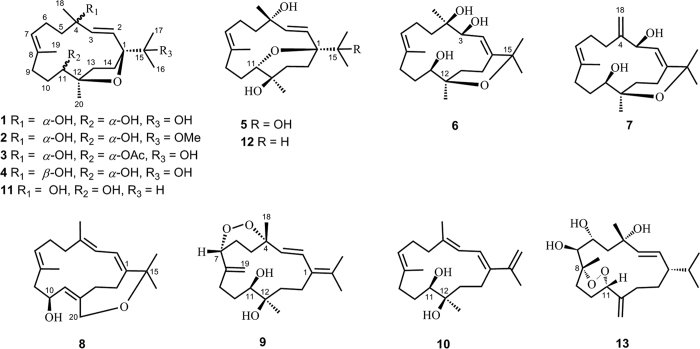
Structures of compounds 1–13.

Samples of *S. trocheliophorum*(dry weight 400 g) were extracted exhaustively with acetone, and the extract was partitioned between water and Et_2_O. The Et_2_O solvable fraction was subjected to repeated chromatography as usual work^3^,^4^,^9^,^16^, to afford ten pure metabolites, compounds **1**–**10**(Fig. 1). A preliminary NMR analysis revealed that all the new molecules shared the same cembrane skeleton. Among them, the known compound was readily identified as sarglaucol(**10**)^19^ by comparison of its spectral data and [*α*]_D_ values with those reported in the literatures.

**Figure 2:**
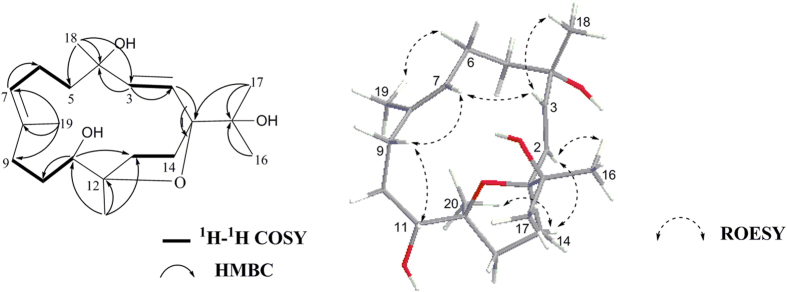
Selected key COSY, HMBC and ROESY correlations for compound 1.

**Figure 3:**
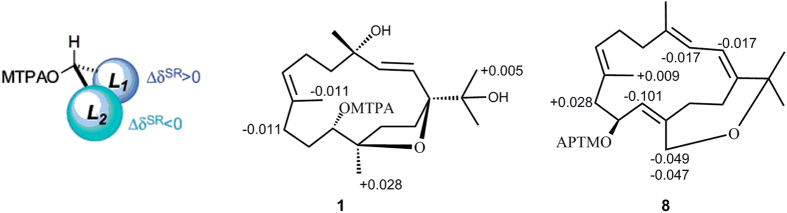
The Δ*δ*^SR^ [Δ(*δ*_S_-*δ*_R_)] data for the MTPA esters of compounds 1 and 8.

The HRESIMS of sarcophytrol M(**1**) established the molecular formula C_20_H_34_O_4_. ^1^H and ^13^C NMR spectra of **1**(Tables 1 and 2) were reminiscent of a known cembranoid,(2*E*,7*E*)-4,11-dihydroxy-1,12-oxidocembra-2,7-diene(**11**), previously isolated from *Sinularia ovispiculata*^20^. The distinct difference between them was the presence of a hydroxyl at C-15(*δ*_C_ 72.4) in **1**, which was further confirmed by the observation of HMBC correlations from H_3_-16(*δ*_H_ 1.12)/H_3_-17(*δ*_H_ 1.06) to C-15(Fig. 2). Similar to **11**, a *trans*-disubstituted olefin at C-2/C-3 was recognized by the doublet coupling constant(15.4 Hz) of H-2(*δ*_H_ 5.62) and H-3(*δ*_H_ 5.92), while the chemical shift of Me-19(*δ*_C_ < 20 ppm) indicated the 7*E* assignment in **1**^20^,^21^. The relative configurations of the stereogenic centers were determined by NOE relationships(Fig. 2), which exhibited similar key cross-peaks to those in the cembranoids sinulariols D and F^7^. The NOE correlation between H-3 and H-11(*δ*_H_ 3.54) implied that the *trans*-disubstituted olefin and the hydroxyl group at C-11 were hind the same face toward the five-membered ring. Thus, Me-20 was oriented to the same face with the isopropyl group, and tentatively assigned to be *α*-oriented. In addition, the significant NOE correlations of H-2/H_3_-16 and H-2/H-14b(*δ*_H_ 1.67) suggested that H-2 and the isopropyl group to be on the same side of **1**. The *trans*-disubstituted olefin group favoring ‘up’ or ‘down’-orientation toward the 14-membered ring, and thus H-3 was oriented to ‘up’ face. The configuration of H-11 was assigned as *β* mainly on the basis of the aforementioned strong NOE correlation between H-11 and H-3 and the very weak NOE correlation between H-11 and H_3_-20(*δ*_H_ 1.11). According to the NOE correlation from H-3 to H_3_-18(*δ*_H_ 1.27) and the lack of correlation from H-2 to H_3_-18, we tentatively assigned the configuration of H_3_-18 to *β* face. To obtain its absolute configuration, two aliquots of compound **1** were treated with(*R*)- and(*S*)-*α*-methoxy-*α-*trifluoromethylphenyl acetyl(MTPA) chlorides to obtain the(*S*)- and(*R*)-esters, respectively. Analysis of Δ*δ*^**SR**^ values(*δ*_*S*_ - *δ*_*R*_) observed for the signals of the protons close to 11-OH(Fig. 3) indicated the *S* configuration at this carbon. Consequently, the absolute configuration of compound **1** was determined as(1*S*, 4*S*, 11*S*, 12*R*).

Sarcophytrol N(**2**) has a molecular formula of C_21_H_36_O_4_, as established by HRESIMS and NMR data, 14 mass units more than that of **1**. The ^1^H NMR data of **2**(Tables 1 and 2) were closely reminiscent to those of **1**, except for the newly appeared signal at *δ*_H_ 3.23(3H, s), which suggested the presence of an additional methoxyl group in **2**, consistent with one carbon resonance at *δ*_C_ 50.6(q) in its ^13^C NMR spectrum. The introduction of the methoxyl group in **2** resulted in the significant downfield shift of C-15 from *δ*_C_ 72.4(s) in **1** to 78.4(s) in **2** and the upfield shifts of C-16/C-17. Furthermore, the methoxyl group was secured at C-15 by a diagnostic HMBC correlation from methoxyl group to C-15. Furthermore, ROESY correlations of compound **2** were similar with those of **1**. On the basis of above evidences, compound **2** was identified as 15-methoxyl derivative of **1**.

Sarcophytrol P(**3**) possesses a molecular formula of C_22_H_36_O_5_ as determined by HRESIMS data, 42 mass units more than that of **1**. The NMR spectroscopic features of **3**(Tables 1 and 2) mostly resembled those of **1**. In fact, the only difference was at C-11 position, where the hydroxyl group(*δ*_H_ 3.54; *δ*_C_ 76.4) in **1** was replaced by an acetyl(*δ*_H_ 5.09, 2.04; *δ*_C_ 77.3, 21.3, 171.0) in **3**. A detailed 2D NMR analysis further confirmed the planar structure of compound **3**. As the patterns of ROESY correlations of **3** were similar with those of **1**, accordingly the structure of **3** was established as the 11-acetyl derivative of **1**. The chemical conversion of **1** into **3** by simple acetylation in Ac_2_O/pyridine further indicated the absolute configuration of **3** is the same as **1**.

Sarcophytrol P(**4**) was isolated as an optically active colorless oil with molecular formula C_20_H_34_O_4_, the same as **1**. The NMR spectra of **4**(Tables 1 and 2) were strongly reminiscent of those of **1** and a careful 2D NMR analysis suggested that they share the same gross structure. In fact, the only difference was found in the segment from C-2 to C-5, where ^13^C NMR chemical shifts of C-3, C-4 and C-5 in **4** were all upfield shifted while that of *γ*-carbon C-2 was downfield shifted with respect of **1**. This evidence clearly suggested that the relative configuration of the hydroxyl group at C-4 of **4** was different from that of **1**. The NOE interactions between H-2(*δ*_H_ 5.55) and H_3_-18(*δ*_H_ 1.33) in **1** rather than H-3(*δ*_H_ 6.07) and H_3_-18 in **4** confirmed this assignment. Herein, **4** was 4-epimer of **1**.

Sarcophytrol Q(**5**) possesses the same molecular formula C_22_H_36_O_5_ as **4**, established by HRESIMS, whereas its NMR data(Tables 1 and 2) were almost identical to those of sinulariol Z(**12**)^22^. The distinction was attributed to the NMR data of **5** presenting an extra hydroxyl group at C-15, which was further confirmed by the observation of HMBC correlations from H_3_-16(*δ*_H_ 1.12)/H_3_-17(*δ*_H_ 1.07) to C-15(*δ*_C_ 74.6). Due to the presence of the 15-OH, ^13^C NMR chemical shifts of C-15, C-16, C-17, and C-1 were all downfield shifted reasonably while those of γ-carbons 2 and 14 were upfield shifted with respect of those of **12**. Furthermore, the significant NOE interactions between H-3(*δ*_H_ 5.98) and H-11(*δ*_H_ 3.34) and the lack of NOE correlations of H-11/H_3_-16(*δ*_H_ 1.12), H-11/H_3_-17(*δ*_H_ 1.07), H-11/H_3_-20(*δ*_H_ 1.16) confirmed the isopropyl group and H_3_-20 were in opposite face against H-11 in the hexatomic ring. Additional strong NOE interaction between H-3 and H_3_-18 indicated H_3_-18 at the same side as H-3 in the case of **5**. Thus, **5** had the same relative configuration relationships as those of **12**, which were further confirmed by the closely identical spectral data for the segment C-3–C-13.

Sarcophytrol R(**6**) was obtained as a colorless, optically active oil {[*α*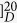 − 13.3(*c* 0.06, MeOH)}. HRESIMS and ^13^C NMR spectra analysis established the molecular formula of **6** as C_20_H_34_O_4_. Thus, four degrees of unsaturation were determined for **6**. The NMR data(Tables 1 and 2) revealed the presence of two trisubstituted double bonds(*δ*_H_ 5.36, *δ*_C_ 146.33, 121.54; *δ*_H_ 5.30, *δ*_C_ 126.73, 135.48), which accounted for two degrees of unsaturation. The remaining two degrees of unsaturation strongly indicated that **6** has a bicyclic structure with a carbocyclic ring bridged by oxygen. The ^13^C NMR spectrum of **6** contained five oxygenated carbon signals at *δ*_C_ 74.04(CH), 76.80(C), 69.49(CH), 75.32(C) and 74.44(C), which were assigned by 2D NMR spectrum to C-3, C-4, C-11, C-12 and C-15, respectively. In order to define the carbon atoms linked to these functionalities, 2D NMR experiments(in DMSO-*d*_6_) of **6** were measured. The formation of an ether bridge across C-12 and C-15 was deduced from the observation of OH-3(*δ*_H_ 4.31), OH-4(*δ*_H_ 3.95) and OH-11(*δ*_H_ 4.10). The location of these three –OH groups are determined by the ^1^H-^1^H COSY correlations of H-3(*δ*_H_ 3.90) with OH-3 and H-11(*δ*_H_ 3.40) with OH-11, and the HMBC correlations from OH-4 to C-3(*δ*_C_ 72.9), C-4(*δ*_C_ 75.1), C-5(*δ*_C_ 38.1).

**Figure 4:**
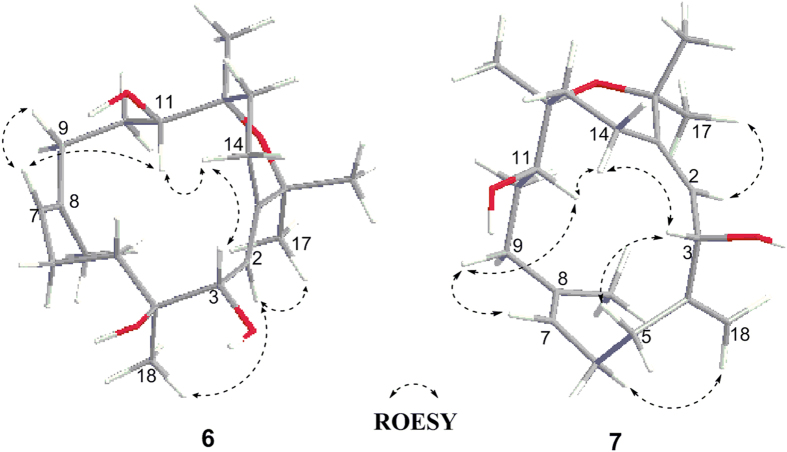
Selected key ROESY correlations for compounds 6 and 7.

The *E* geometry of the Δ^1^ and Δ^9^ double bonds was deduced by the ROESY correlations of H-2(*δ*_H_ 5.36)/H_3_-17(*δ*_H_ 1.31) and of H-7(*δ*_H_ 5.30)/H_2_-9(*δ*_H_ 2.12)(Fig. 4). Moreover, H-14a(*δ*_H_ 2.85) correlated with H-3(*δ*_H_ 4.19) and H-11(*δ*_H_ 3.57), suggesting H-3 and H-11 to be on the same side of **6**. When H-3 was assigned tentatively as *β*-orientation, H-11 was accordingly oriented in *β*-face. The presence of the cross-peak between H-2 and H_3_-18(*δ*_H_ 1.27) in combination with the absence of the correlations of H-3/H-2 and H-3/H_3_-18 in the ROESY spectrum, clarified H_3_-18 and H-2 to be oriented in opposite to H-3. Finally, the *α*-oriented H_3_-20 was tentatively deduced by the absence of correlations between H_3_-20 and H-11.

Sarcophytrol S(**7**) was isolated as an optically active, colorless oil {[*α*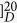 − 39.3(*c* 0.07, MeOH)}. Its molecular formula, C_20_H_32_O_3_, was established by HRESIMS, 18 units less than that of **6**. Careful comparison of NMR data of **7** and **6**(Tables 1 and 2) revealed that the former differs from the latter only by the presence of a terminal methylene group(*δ*_H_ 5.13, 4.93; *δ*_C_ 111.37, 147.92) in **7** instead of a methyl(*δ*_H_ 1.27; *δ*_C_ 22.91) and a oxygenated quarternary carbon(*δ*_C_ 76.8) in **6**, in agreement with 18 mass units difference between them, while the rest of the molecules was the same. Base on the HMBC correlations between H_2_-18(*δ*_H_ 5.13, 4.93) and C-3(*δ*_C_ 75.24) and C-5(*δ*_C_ 28.32), the terminal methylene group was located at C-4. Moreover, ^1^H-^1^H COSY, HSQC, and HMBC experiments allowed the unambiguous definition of the structure of **7**. Analogously to **6**, the relative stereochemistry of three chiral centers at C-3, C-11 and C-12 was elucidated to be the same as those of **6** by the ROESY experiments(Fig. 4).

The HRESIMS of sarcophytrol T(**8**) established the molecular formula C_20_H_30_O_2_, indicating six degrees of unsaturation. The presence of four double bonds(*δ*_H_ 6.09, *δ*_C_ 140.2, 123.5; *δ*_H_ 5.66, *δ*_C_ 137.2, 123.4; *δ*_H_ 5.05, *δ*_C_ 125.5, 133.5; *δ*_H_ 5.07, *δ*_C_ 124.8, 145.9) accounted for four degrees of unsaturation. The remaining two degrees of unsaturation strongly indicated that **8** has a bicyclic structure. A HMBC relationship between H_2_-20(*δ*_H_ 4.25 and 4.07) and C-15(*δ*_C_ 78.9) ascertained that an ether bridge formed across C-20 and C-15. Key NOE correlations from H-3(*δ*_H_ 5.66) to H-5b(*δ*_H_ 1.98) and H-14a(*δ*_H_ 2.60) and from H-2(*δ*_H_ 6.09) to H_3_-18(*δ*_H_ 1.75) and H_3_-17(*δ*_H_ 1.35) suggested the olefinic geometries were assigned to 1*E* and 3*E*. The chemical shift of Me-19(*δ*_C_ < 20 ppm) indicated the 7*E* assignment^21^. The additional hydroxylation at C-10 was supported by the ^1^H-^1^H COSY correlations of H-10(*δ*_H_ 4.26)/H-11(*δ*_H_ 5.07) and H-10/H-9(*δ*_H_ 2.40 and 2.16). Since compound **8** contained a secondary alcohol at C-10, its absolute configuration of C-10 was determined to be *S*, by applying the modified Mosher’s method following the same protocol as used for **1**(Fig. 3).

**Figure 5:**
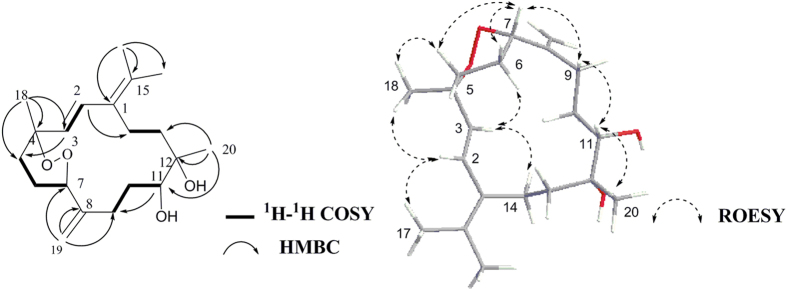
Selected key COSY, HMBC and ROESY correlations for compound 9.

Sarcophytrol U(**9**), a colorless oil, had a molecular formula of C_20_H_32_O_4_ established by HRESIMS(*m/z* 359.2199 [M + Na]^+^) and NMR data, indicating five degrees of unsaturation. The presence of a secondary and a tertiary hydroxyl group was clearly deduced from NMR signals at *δ*_C_ 76.9(d), 74.4(s) and *δ*_H_ 3.44(1H, dd, *J* = 10.2, 2.2 Hz, H-11). One additional terminal double bond was inferred by ^13^C NMR signals at *δ*_C_ 145.9(C) and 119.1(CH_2_). The conjugated olefinic group was also evident by four sp^2^ carbon signals in the ^13^C NMR spectrum at *δ*_C_ 130.3(s), 129.0(d), 132.8(d), and 123.0(s), and two olefinic doublets in the ^1^H NMR spectrum at *δ*_H_ 6.33(1H, d, *J* = 16.6 Hz, H-2) and 5.97(1H, d, *J* = 16.6 Hz, H-3). Detailed analysis of the 2D spectra(Fig. 5), allowed assigning all the chemical shifts in the NMR spectra, which led to the cembrane skeleton of **9**. An *exo*-CH_2_, the conjugated olefinic groups and the 14-member ring of cembrane skeleton accounted for four degrees of unsaturation. As a consequence, there must be two oxygen atoms unassigned had to be ascribed to a peroxide bridge that linked at C-4 and C-7, respectively, to complete the required unsaturation degrees of five. In addition, the analysis of the NMR data of **9**, in comparison with that of a known cembranoid,(1*S*,2*E*,4*S*,6*R*,7*S*,8*R*,11*S*)-8,11-epidioxy-2,12(20)-cembradiene-4,6,7-triol(**13**), previously reported from Greek tobacco^23^, confirmed the partial structure from C-4-C-9. Thus, the gross planar structure of **9** was determined as a 4,7-epidioxy-15(1),2,8(19)-cembratriene-11,12-diol.

The relative stereochemistry of the chiral centers at C-4, C-7, C-11 and C-12 was established by a ROESY experiment running on **9**. In MM2 energy-minimized conformation(Fig. 5), H-7 was suggested to be axial position toward the hexatomic ring(*β*-orientation). The diagnostic correlations between H-7(*δ*_H_ 4.71) and H-5a(*δ*_H_ 1.87) as well as H_3_-18(*δ*_H_ 1.28) and H-5a suggested H-5a and H_3_-18 were determined as axial and equatorial position, respectively(*β*-orientation). The ROESY correlation of H-11(*δ*_H_ 3.44) with H_3_-20(*δ*_H_ 1.28) suggested the H-11 and H_3_-20 are in the same face, which was compatible with that of the co-exist cembranoid sarglaucol(**10**)^19^. However, the relationship of H-7 and H-11 cannot be determined from the ROESY spectrum.

In conclusion, nine new cembranoids, sarcophytrols M–U(**1**–**9**), were isolated from the South China Sea soft coral *Sarcophyton trocheliophorum*, along with one known related cembranoid **10**. Among them, the characteristic chemical features of them are the diverse types of cyclized rings: furan rings possessed by sarcophytrols M-P(**1**–**4**), pyran rings formed in sarcophytrols Q-S(**5**–**7**), while oxepane and peroxyl rings appeared in sarcophytrols T(**8**) and U(**9**), respectively. In addition, sarcophytrols R and S(**6** and **7**) share a rare bicyclic skeleton of the decaryiol-type. These group of diterpenes were first reported from the same genus of soft coral *S. decaryi*^11^, and described so far only from the species of soft corals *Lobophytum* sp.^12^. The co-isolation of these diterpenes is an example of the productivity of the title animal.

In light of a wide range of biological activities and pharmacological properties of cembranoids^13^, we performed *in vitro* investigation of inhibitory activity against human protein tyrosine phosphatase 1B(PTP1B) enzyme, a promising drug target for the treatment of type 2 diabetes and obesity^17^, for compounds **1**–**10**, since the previously reported cembranoids from the title animals displayed significant PTP1B inhibitory activity^3^,^4^,^16^. Unfortunately, the bioassay result showed that none of the tested compounds exhibited interesting PTP1B inhibitory activities. In addition, the antitumor and antibacterial activities were also tested for compounds **1**–**10**. However, they exhibited neither cytotoxicities against the human tumor cell lines HL-60 and K-562, nor antibacterial activity against *Pseudomonas aeruginosa.*

## Methods

### General experimental procedures

Optical rotations were measured on a Perkin-Elmer 341 polarimeter. IR spectra were recorded on a Perkin-Elmer 577 spectrometer with KBr disks. HRESIMS spectra were recorded on a Waters-Micromass Q-TOF Ultima Global electrospray mass spectrometer. NMR spectra were measured on a Bruker Avance III 500 and Varian INOVA 600 spectrometers with the residual CHCl_3_(*δ*_H_ 7.26 ppm, *δ*_C_ 77.0 ppm) as internal standard. Chemical shifts are expressed in *δ*(ppm) and coupling constants(*J*) in Hz. ^1^H and ^13^C NMR assignments were supported by ^1^H-^1^H COSY, HSQC, and HMBC experiments. Commercial Silica gel(Qing Dao Hai Yang Chemical Group Co., 200–300 and 400–600 mesh), C18 reversed-phase silica gel(150–200 mesh, Merck) and Sephadex LH-20(Amersham Biosciences) were used for column chromatography. Reversed phase HPLC(Agilent 1100 series liquid chromatography using a VWDG1314A detector at 210 nm and a semi-preparative ODS-HG-5 [5 μm, 10 mm(i.d.) × 25 cm] column was also employed. Pre-coated silica gel GF_254_ plates(Qing Dao Hai Yang Chemical Group Co. Ltd. Qingdao, People’s Republic of China) were used for analytical thin-layer chromatography(TLC). All solvents used were of analytical grade(Shanghai Chemical Reagents Company, Ltd.).

### Animal materials

The soft corals *S. trocheliophorum* was collected by scuba at Yalong Bay, Hainan Province, China, in February 26, 2006, at a depth of −15 to −20 m, and identified by Professor R.-L. Zhou of South China Sea Institute of Oceanology, Chinese Academy of Sciences. The voucher sample is deposited at the Shanghai Institute of Materia Medica, CAS, under registration No. YAL-4.

### Extraction and isolation

The lyophilized bodies of *S. trocheliophorum*(400 g, dry weight) were minced into pieces and exhaustively extracted with Me_2_CO at room temperature(3 × 1 L). The solvent-free Me_2_CO extract was partitioned between Et_2_O and H_2_O. The organic phase was evaporated under reduced pressure to give a dark brown residue(16 g), which was subjected to Si gel column chromatography(CC) and eluted with petroleum ether(PE) in acetone(0–100%, gradient) to yield 14 fractions(A–M). These fractions were subjected to repeated chromatography as usual work^3^,^4^,^9^,^16^, to afford ten pure metabolites: **1**(5.7 mg), **2**(4.8 mg), **3**(4.2 mg), **4**(3.6 mg), **5**(6.7 mg), **6**(3.1 mg), **7**(2.0 mg), **8**(5.6 mg), **9**(1.0 mg), **10**(2.1 mg).

### Chemical structure data

All investigated compounds were ≥95% pure(HPLC, wavelength = 210 nm).

The NMR spectra of the compounds are provided in the Supporting Information.

**Sarcophytrol M(1**): colorless oil; 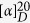 − 60.8(*c* 0.17, MeOH). IR(KBr) *ν*_max_ 3438, 2936, 1645, 1098, 950, 754 cm^−1^; for ^1^H and ^13^C NMR data, see Tables 1 and 2; HRESIMS *m/z* 361.2356 [M + Na]^+^(calcd for 361.2355, C_20_H_34_O_4_Na).

**Sarcophytrol N(2)**: colorless oil; 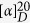 − 120.0(*c* 0.15, MeOH). IR(KBr) *ν*_max_ 3435, 2922, 1648, 1116, 954 cm^−1^; for ^1^H and ^13^C NMR data, see Tables 1 and 2; HRESIMS *m/z* 375.2504 [M + Na]^+^(calcd for 375.2511, C_21_H_36_O_4_Na).

**Sarcophytrol O(3)**: colorless oil; 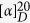 − 30.0(*c* 0.10, MeOH). IR(KBr) *ν*_max_ 3433, 2932, 1733, 1638, 1262, 1024 cm^−1^; for ^1^H and ^13^C NMR data, see Tables 1 and 2; HRESIMS *m/z* 403.2458 [M + Na]^+^(calcd for 403.2455, C_22_H_36_O_5_Na).

**Sarcophytrol P(4)**: colorless oil; 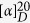 + 24.4(*c* 0.10, MeOH). IR(KBr) *ν*_max_ 3441, 2942, 1648, 1262, 1098, 802 cm^-1^; for ^1^H and ^13^C NMR data, see Tables 1 and 2; HRESIMS *m/z* 361.2345 [M + Na]^+^(calcd for 361.2355, C_20_H_34_O_4_Na).

**Sarcophytrol Q(5)**: colorless oil; 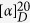 + 45.9(*c* 0.22, MeOH). IR(KBr) *ν*_max_ 3458, 2950, 1650, 1232, 1028, 756 cm^−1^; for ^1^H and ^13^C NMR data, see Tables 1 and 2; HRESIMS *m/z* 361.2354 [M + Na]^+^(calcd for 361.2355, C_20_H_34_O_4_Na).

**Sarcophytrol R(6)**: colorless oil; 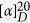 − 13.3(*c* 0.06, MeOH). IR(KBr) *ν*_max_ 3455, 2932, 1653, 1255, 768 cm^−1^; ^1^H and ^13^C NMR data, see Tables 1 and 2; HRESIMS *m/z* 361.2346 [M + Na]^+^(calcd for 361.2355, C_20_H_34_O_4_Na).

**Sarcophytrol S(7)**: colorless oil; 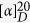 − 39.3(*c* 0.07, MeOH). IR(KBr) *ν*_max_ 3437, 2947, 1633, 1198, 879 cm^−1^; for ^1^H and ^13^C NMR data, see Tables 1 and 2; HRESIMS *m/z* 343.2252 [M + Na]^+^(calcd for 343.2249, C_20_H_32_O_3_Na).

**Sarcophytrol T(8)**: colorless oil; 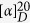 − 35.4(*c* 0.08, MeOH); UV(MeOH) λ_max_ 242 nm. IR(KBr) *ν*_max_ 3418, 2962, 1568, 1262, 782 cm^−1^; for ^1^H and ^13^C NMR data, see Tables 1 and 2; HRESIMS *m/z* 325.2141 [M + Na]^+^(calcd for 325.2144, C_20_H_30_O_2_Na).

**Sarcophytrol U(9)**: colorless oil; 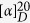 + 29.0(*c* 0.10, MeOH); UV(MeOH) λ_max_ 238 nm. IR(KBr) *ν*_max_ 3425, 2955, 1753, 1662, 778 cm^−1^; for ^1^H and ^13^C NMR data, see Tables 1 and 2; HRESIMS *m/z* 359.2199 [M + Na]^+^(calcd for 359.2198, C_20_H_32_O_4_Na).

**Preparation of(*****S*****)- and(*****R*****)-MTPA Esters of 1.** The **1***S* derivative was obtained by treating **1**(2.0 mg) with(*R*)-MTPA-Cl in dry pyridine for ca. 16 h under stirring at room temperature. The reaction mixture was purified by CC(silica gel) to afford pure **1***S*(1.4 mg). In a similar manner, **1***R*(1.2 mg) was prepared from(*S*)-MTPA-Cl.

**1***S*: Selected ^1^H NMR(CDCl_3_, 400 MHz) *δ* 5.997(1H, d, *J* = 15.4 Hz, H-3), 5.657(1H, d, *J* = 15.4 Hz, H-2), 5.504(1H, dd, *J* = 10.4, 4.3 Hz, H-7), 5.273(1H, d, *J* = 9.3 Hz, H-11), 1.673(3H, s, Me-19), 1.284(3H, s, Me-18), 1.122(3H, s, Me-16), 1.100(3H, s, Me-20), 1.072(3H, s, Me-17).

**1***R*: Selected ^1^H NMR(CDCl_3_, 400 MHz) *δ* 6.000(1H, d, *J* = 15.4 Hz, H-3), 5.656(1H, d, *J* = 15.4 Hz, H-2), 5.515(1H, dd, *J* = 11.1, 4.3 Hz, H-7), 5.288(1H, d, *J* = 9.9 Hz, H-11), 1.684(3H, s, Me-19), 1.285(3H, s, Me-18), 1.117(3H, s, Me-16), 1.072(3H, s, Me-20), 1.072(3H, s, Me-17).

**Preparation of(*****S*****)- and(*****R*****)-MTPA Esters of 8.** The **8***S* derivative was obtained by treating **8**(2.0 mg) with(*R*)-MTPA-Cl in dry pyridine for ca. 16 h under stirring at room temperature. The reaction mixture was purified by CC(silica gel) to afford pure **8***S*(1.4 mg). In a similar manner, **8***R*(1.2 mg) was prepared from(*S*)-MTPA-Cl.

**8***S*: Selected ^1^H NMR(CDCl_3_, 400 MHz) *δ* 6.111(1H, d, *J* = 10.9 Hz, H-2), 5.642(1H, d, *J* = 10.9 Hz, H-3), 5.591(1H, m, H-10), 5.098(1H, dd, *J* = 9.9, 5.2 Hz, H-7), 4.969(1H, d, *J* = 9.4 Hz, H-11), 4.206(1H, dd, *J* = 16.8, 1.4 Hz, Ha-20), 4.008(1H, dd, *J* = 16.8, 2.2 Hz, Hb-20), 1.767(3H, s, Me-18), 1.458(3H, s, Me-19), 1.379(3H, s, Me-16), 1.357(3H, s, Me-17).

**8***R*: Selected ^1^H NMR(CDCl_3_, 400 MHz) *δ* 6.128(1H, d, *J* = 10.9 Hz, H-2), 5.659(1H, d, *J* = 10.9 Hz, H-3), 5.626(1H, dt, *J* = 10.2, 3.8 Hz, H-10), 5.070(1H, dd, *J* = 9.9, 5.2Hz, H-7), 5.070(1H, d, *J* = 9.4 Hz, H-11), 4.255(1H, dd, *J* = 16.8, 1.1 Hz, Ha-20), 4.055(1H, dd, *J* = 16.8, 1.9 Hz, Hb-20), 1.769(3H, s, Me-18), 1.449(3H, s, Me-19), 1.386(3H, s, Me-16), 1.357(3H, s, Me-17).

## Additional Information

**How to cite this article:** Liang, L.-F. *et al*. New Bicyclic Cembranoids from the South China Sea Soft Coral *Sarcophyton trocheliophorum. Sci. Rep.* **7**, 46584; doi: 10.1038/srep46584(2017).

**Publisher's note:** Springer Nature remains neutral with regard to jurisdictional claims in published maps and institutional affiliations.

## Supplementary Material

Supplementary Information

## Figures and Tables

**Table 1:** ^1^H NMR spectroscopic data for compounds 1–9^a^.

No.	1	2	3	4	5	6	7	8	9
1									
2	5.62 d(15.4)	5.68 d(15.6)	5.64 d(15.4)	5.55 d(15.7)	5.27 d(16.0)	5.46 dd(7.3, 1.4)	5.36 d(7.0)	6.09 d(10.9)	6.33 d(16.6)
3	5.92 d(15.4)	5.88 d(15.6)	6.00 d(15.4)	6.07 d(15.7)	5.98 d(16.0)	4.84 d(7.3)	4.19 d(7.0)	5.66 d(10.9)	5.97 d(16.6)
4									
5	1.82 m	1.83 ddd(13.4 11.5, 1.9)	1.82 m	1.82 m	1.90 m	2.35 m	1.92 m	2.22 m	1.99 ddd(13.6, 4.8, 2.5)
	1.57 m	1.59 ddd(13.4, 13.2, 8.1)	1.58 m	1.57 m	1.60 m	2.30 m	1.69 m	1.98 m	1.87 dd(13.6, 4.8)
6	2.26 t(11.4)	2.27 m	2.28 t(11.6)	2.27 t(11.3)	2.20 m	2.36 m	2.17 m	2.25 m	2.15 m
	2.15 m	2.16 m	2.49 m	2.47 m		2.21 m		2.03 m	1.54 m
7	5.19 dd(10.9, 3.8)	5.20 dd(11.3, 4.5)	5.44 dd(10.9, 4.0)	5.21 dd(9.9, 4.5)	5.21 t(7.3)	5.32 t(6.6)	5.30 t(5.9)	5.05 m	4.71 dd(12.0, 2.9)
8									
9	2.09 m	2.10 dd(12.6, 2.4)	2.09 m	2.10 m	2.19 m	2.11 dd(13.2, 3.6)	2.12 m	2.40 m	2.42 dd(13.4, 6.1)
		2.07 m			2.01 m	2.11 dt(13.2, 4.0)		2.16 m	2.13 m
10	1.89 dt(13.3, 2.5)	2.14 m	1.88 dt(13.2, 2.4)	1.88 m	1.90 m	1.74 ddt(14.0, 3.6, 1.6)	1.85 m	4.26, dd(20.1, 3.0)	2.00 m
	1.35 m	1.83 ddd(13.2, 8.1, 1.9)			1.39 m	1.20 m		1.25 m	1.41 m
11	3.54 d(9.5)	3.53 d(9.6)	5.09 d(9.6)	3.54 d(9.4)	3.34 t(4.06)	3.31 d(10.0)	3.57 d(10.5)	5.07 m	3.44 dd(10.2, 2.2)
12									
13	1.77 m	1.74 m	1.79 m	1.79 m	1.70 m	2.12 m	2.17 m	2.79 dd(19.6, 11.2)	1.62 m
		1.72 m			1.51 m	1.87 m	1.91 m	2.13 m	
14	2.41 td(12.1, 7.7)	2.39 m	2.45 td(12.1, 7.6)	2.41 td(12.0, 7.7)	2.19 m	2.49 ddd(17.5, 11.7, 10.0)	2.85 dd(17.8, 9.8)	2.60 ddd(13.6, 10.2, 8.2)	2.49 m
	1.67 m	1.72 m	1.66 m	1.67 m	1.53 m	2.25 m	2.40 dd(17.8, 9.0)	2.35 t(12.1)	1.93 m
15									
16	1.12 s	1.15 s	1.15 s	1.13 s	1.12 s	1.34 s	1.38 s	1.38 s	1.77 s
17	1.06 s	1.06 s	1.07 s	1.10 s	1.07 s	1.28 s	1.31 s	1.35 s	1.73 s
18	1.27 s	1.30 s	1.27 s	1.33 s	1.36 s	5.13 s	1.27 s	1.75 s	1.28 s
						4.93 s			
19	1.68 s	1.68 s	1.67 s	1.72 s	1.65 s	1.60 s	1.61 s	1.38 s	5.11 d(1.8)
									5.09 brs
20	1.11 s	1.09 s	1.12 s	1.12 s	1.16 s	1.04 s	1.06 s	4.25 d(16.4)	1.28 s
								4.07 dd(16.4, 1.5)	
OMe		3.23 s							
OAc			2.04 s						

^a^Spectra measured at 500 MHz in CDCl_3_.

**Table 2:** ^13^C NMR spectroscopic data for compounds 1–9^a^, 11 and 12.

No.	11^[12]^	1	2	3	4	5	12^[22]^	6	7	8	9
1	88.2, s	91.3, s	90.8, s	91.8, s	91.0, s	81.0, s	77.9, s	141.5, s	146.3, s	140.2, s	130.3, s
2	129.0, d	128.1, d	129.4, d	127.9, d	130.4, d	122.1, d	125.0, d	123.5, d	121.5, d	123.5, d	129.0, d
3	137.7, d	138.4, d	137.3, d	138.6, d	137.0, d	142.3, d	140.9, d	75.2, d	74.0, d	123.4, d	132.8, d
4	74.4, s	74.5, s	74.5, s	74.5, s	72.5, s	74.3, s	74.2, s	147.9, s	76.8, s	137.2, s	79.4, s
5	42.9, t	43.9, t	43.6, t	43.6, t	41.8, t	44.5, t	44.5, t	28.3, t	36.9, t	40.0, t	33.1, t
6	28.4, t	24.4, t	24.4, t	24.4, t	24.4, t	23.8, t	23.6, t	22.8, t	21.5, t	26.0, t	24.2, t
7	128.4, d	129.3, d	129.0, d	130.1, d	129.4, d	129.8, d	129.1, d	124.8, d	126.7, d	125.5, d	85.3, d
8	133.6, s	133.5, s	133.5, s	131.9, s	133.3, s	132.8, s	133.1, s	134.6, s	135.5, s	133.5, s	145.9, s
9	34.6, t	35.5, t	35.3, t	35.4, t	35.3, t	38.6, t	37.5, t	34.9, t	34.4, t	50.2, t	31.1, t
10	29.6, t	29.4, t	29.5, t	30.7, t	29.5, t	26.2, t	25.8, t	26.5, t	26.7, t	64.8, d	31.0, t
11	76.2, d	76.4, d	76.4, d	77.3, d	76.2, d	75.0, d	74.8, d	68.8, d	69.5, d	124.8, d	76.9, d
12	84.6, s	85.4, s	85.4, s	84.2, s	85.5, s	70.6, s	70.6, s	74.8, s	75.3, s	145.9, s	74.4, s
13	36.6, t	36.6, t	36.2, t	34.8, t	36.6, t	37.0, t	37.2, t	28.7, t	28.9, t	32.0, t	37.8, t
14	35.2, t	31.0, t	31.9, t	30.7, t	30.9, t	28.1, t	30.3, t	18.9, t	20.4, t	24.2, t	25.1, t
15	39.2, d	72.4, s	78.4, s	72.4, s	72.2, s	74.6, s	39.5, d	74.3, s	74.4, s	78.9, s	123.0, s
16	18.4, q	24.4, q	20.2, q	24.3, q	24.4, q	23.7, q	17.1, q	31.2, q	30.5, q	24.0, q	20.7, q
17	17.6, q	26.0, q	21.9, q	25.9, q	25.9, q	24.4, q	17.0, q	29.5, q	29.2, q	27.9, q	20.5, q
18	29.3, q	28.4, q	28.7, q	28.0, q	28.7, q	27.7, q	29.0, q	111.4, t	22.9, q	16.5, q	25.5, q
19	16.7, q	16.3, q	16.4, q	16.0, q	16.4, q	14.8, q	14.8, q	15.6, q	16.8, q	16.1, q	119.1, t
20	20.0, q	19.4, q	19.3, q	20.6, q	19.4, q	19.1, q	19.5, q	22.6, q	22.6, q	62.4, t	25.9, q
OMe			50.6, q								
OAc				21.3, q							
				171.0, s							

^a^Spectra measured at 125 MHz in CDCl_3_.
