# Supplementary material for: New Bicyclic Cembranoids from the South China Sea Soft Coral Sarcophyton trocheliophorum
Source: Sci Rep. 2017 Apr 21;7:46584. doi: 10.1038/srep46584 (PMC5399464; doi:10.1038/srep46584)
Supplement: Supplementary Information [file srep46584-s1.doc]

**Supplementary data**

New Bicyclic Cembranoids from the South China Sea Soft Coral *Sarcophyton trocheliophorum*

Lin-Fu Liang,[[1]](#footnote-2),2,# Wen-Ting Chen,^1,#^ Xu-Wen Li,^1^ He-Yao Wang,^1^ Yue-Wei Guo^1,*^

**Figure S1**. ^1^H NMR spectrum (500 MHz, CDCl_3_) of sarcophytrol M (**1**)

**Figure S2**. ^13^C NMR spectrum (125 MHz, CDCl_3_) of sarcophytrol M (**1**)

**Figure S3**. HMQC spectrum (500 MHz, CDCl_3_) of sarcophytrol M (**1**)

**Figure S4**. HMBC spectrum (500 MHz, CDCl_3_) of sarcophytrol M (**1**)

**Figure S5**. ^1^H-^1^H COSY spectrum (500 MHz, CDCl_3_) of sarcophytrol M (**1**)

**Figure S6**. NOE spectrum (400 MHz, CDCl_3_) of sarcophytrol M (**1**)

**Figure S7**. HRESIMS spectrum of sarcophytrol M (**1**)

**Figure S8**. ^1^H NMR spectrum (500 MHz, CDCl_3_) of sarcophytrol N (**2**)

**Figure S9**. ^13^C NMR spectrum (125 MHz, CDCl_3_) of sarcophytrol N (**2**)

**Figure S10**. HMQC spectrum (500 MHz, CDCl_3_) of sarcophytrol N (**2**)

**Figure S11**. HMBC spectrum (500 MHz, CDCl_3_) of sarcophytrol N (**2**)

**Figure S12**. HRESIMS spectrum of sarcophytrol N (**2**)

**Figure S13**. ^1^H NMR spectrum (500 MHz, CDCl_3_) of sarcophytrol O (**3**)

**Figure S14**. ^13^C NMR spectrum (125 MHz, CDCl_3_) of sarcophytrol O (**3**)

**Figure S15**. HMQC spectrum (500 MHz, CDCl_3_) of sarcophytrol O (**3**)

**Figure S16**. HMBC spectrum (500 MHz, CDCl_3_) of sarcophytrol O (**3**)

**Figure S17**. HRESIMS spectrum of sarcophytrol O (**3**)

**Figure S18**. ^1^H NMR spectrum (500 MHz, CDCl_3_) of sarcophytrol P (**4**)

**Figure S19**. ^13^C NMR spectrum (125 MHz, CDCl_3_) of sarcophytrol P (**4**)

**Figure S20**. HMQC spectrum (500 MHz, CDCl_3_) of sarcophytrol P (**4**)

**Figure S21**. NOE spectrum (400 MHz, CDCl_3_) of sarcophytrol P (**4**)

**Figure S22**. HRESIMS spectrum of sarcophytrol P (**4**)

**Figure S23**. ^1^H NMR spectrum (500 MHz, CDCl_3_) of sarcophytrol Q (**5**)

**Figure S24**. ^13^C NMR spectrum (125 MHz, CDCl_3_) of sarcophytrol Q (**5**)

**Figure S25**. HMQC spectrum (500 MHz, CDCl_3_) of sarcophytrol Q (**5**)

**Figure S26**. HMBC spectrum (500 MHz, CDCl_3_) of sarcophytrol Q (**5**)

**Figure S27**. ^1^H-^1^H COSY spectrum (500 MHz, CDCl_3_) of sarcophytrol Q (**5**)

**Figure S28**. NOE spectrum (400 MHz, CDCl_3_) of sarcophytrol Q (**5**)

**Figure S29**. HRESIMS spectrum of sarcophytrol Q (**5**)

**Figure S30**. ^1^H NMR spectrum (500 MHz, CDCl_3_) of sarcophytrol R (**6**)

**Figure S31**. ^13^C NMR spectrum (125 MHz, CDCl_3_) of sarcophytrol R (**6**)

**Figure S32**. HMQC spectrum (500 MHz, CDCl_3_) of sarcophytrol R (**6**)

**Figure S33**. HMBC spectrum (500 MHz, CDCl_3_) of sarcophytrol R (**6**)

**Figure S34**. ^1^H-^1^H COSY spectrum (500 MHz, CDCl_3_) of sarcophytrol R (**6**)

**Figure S35**. ROESY spectrum (500 MHz, CDCl_3_) of sarcophytrol R (**6**)

**Figure S36**. HRESIMS spectrum of sarcophytrol R (**6**)

**Figure S37**. ^1^H NMR spectrum (500 MHz, CDCl_3_) of sarcophytrol S (**7**)

**Figure S38**. ^13^C NMR spectrum (125 MHz, CDCl_3_) of sarcophytrol S (**7**)

**Figure S39**. HMQC spectrum (500 MHz, CDCl_3_) of sarcophytrol S (**7**)

**Figure S40**. HMBC spectrum (500 MHz, CDCl_3_) of sarcophytrol S (**7**)

**Figure S41**. ^1^H-^1^H COSY spectrum (500 MHz, CDCl_3_) of sarcophytrol S (**7**)

**Figure S42**. ROESY spectrum (500 MHz, CDCl_3_) of sarcophytrol S (**7**)

**Figure S43**. ^1^H NMR spectrum (500 MHz, DMSO) of sarcophytrol S (**7**)

**Figure S44**. ^13^C NMR spectrum (125 MHz, DMSO) of sarcophytrol S (**7**)

**Figure S45**. HMQC spectrum (500 MHz, DMSO) of sarcophytrol S (**7**)

**Figure S46**. HMBC spectrum (500 MHz, DMSO) of sarcophytrol S (**7**)

**Figure S47**. ^1^H-^1^H COSY spectrum (500 MHz, DMSO) of sarcophytrol S (**7**)

**Figure S48**. NOE spectrum (400 MHz, DMSO) of sarcophytrol S (**7**)

**Figure S49**. HRESIMS spectrum of sarcophytrol S (**7**)

**Figure S50**. ^1^H NMR spectrum (500 MHz, CDCl_3_) of sarcophytrol T (**8**)

**Figure S51**. ^13^C NMR spectrum (125 MHz, CDCl_3_) of sarcophytrol T (**8**)

**Figure S52**. HMQC spectrum (500 MHz, CDCl_3_) of sarcophytrol T (**8**)

**Figure S53**. HMBC spectrum (500 MHz, CDCl_3_) of sarcophytrol T (**8**)

**Figure S54**. ^1^H-^1^H COSY spectrum (500 MHz, CDCl_3_) of sarcophytrol T (**8**)

**Figure S55** HRESIMS spectrum of sarcophytrol T (**8**)

**Figure S56**. ^1^H NMR spectrum (500 MHz, CDCl_3_) of sarcophytrol U (**9**)

**Figure S57**. ^13^C NMR spectrum (125 MHz, CDCl_3_) of sarcophytrol U (**9**)

**Figure S58**. HMQC spectrum (500 MHz, CDCl_3_) of sarcophytrol U (**9**)

**Figure S59**. HMBC spectrum (500 MHz, CDCl_3_) of sarcophytrol U (**9**)

**Figure S60**. ^1^H-^1^H COSY spectrum (500 MHz, CDCl_3_) of sarcophytrol U (**9**)

**Figure S61**. ROESY spectrum (500 MHz, CDCl_3_) of sarcophytrol U (**9**)

**Figure S62**. HRESIMS spectrum of sarcophytrol U (**9**)

**Figure S1**. ^1^H NMR spectrum (500 MHz, CDCl_3_) of sarcophytrol M (**1**)

**Figure S2**. ^13^C NMR spectrum (125 MHz, CDCl_3_) of sarcophytrol M (**1**)

**Figure S3**. HMQC spectrum (500 MHz, CDCl_3_) of sarcophytrol M (**1**)

**Figure S4**. HMBC spectrum (500 MHz, CDCl_3_) of sarcophytrol M (**1**)

**Figure S5**. ^1^H-^1^H COSY spectrum (500 MHz, CDCl_3_) of sarcophytrol M (**1**)

**Figure S6**. NOEDIFF spectrum (400 MHz, CDCl_3_) of sarcophytrol M (**1**)


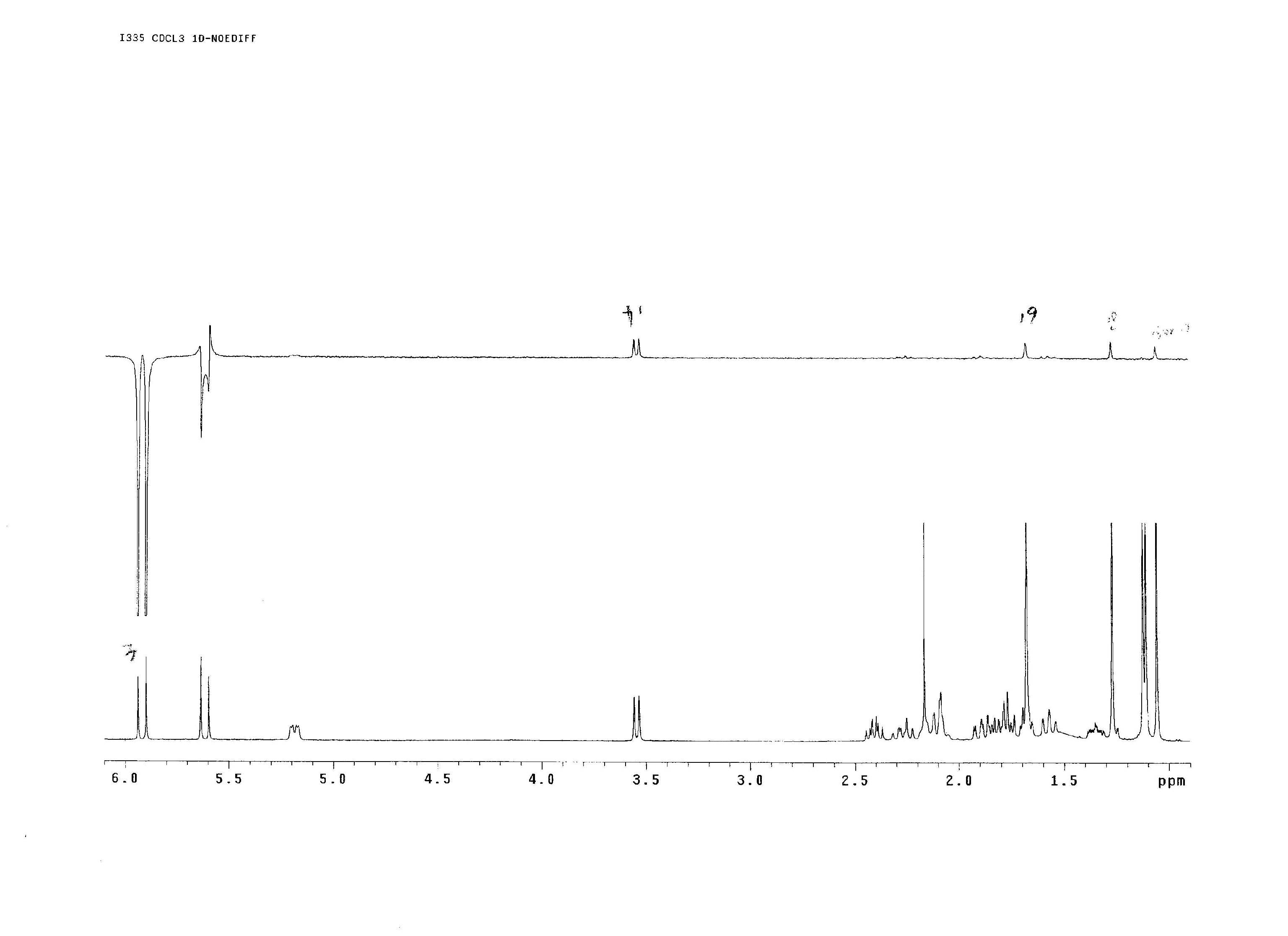


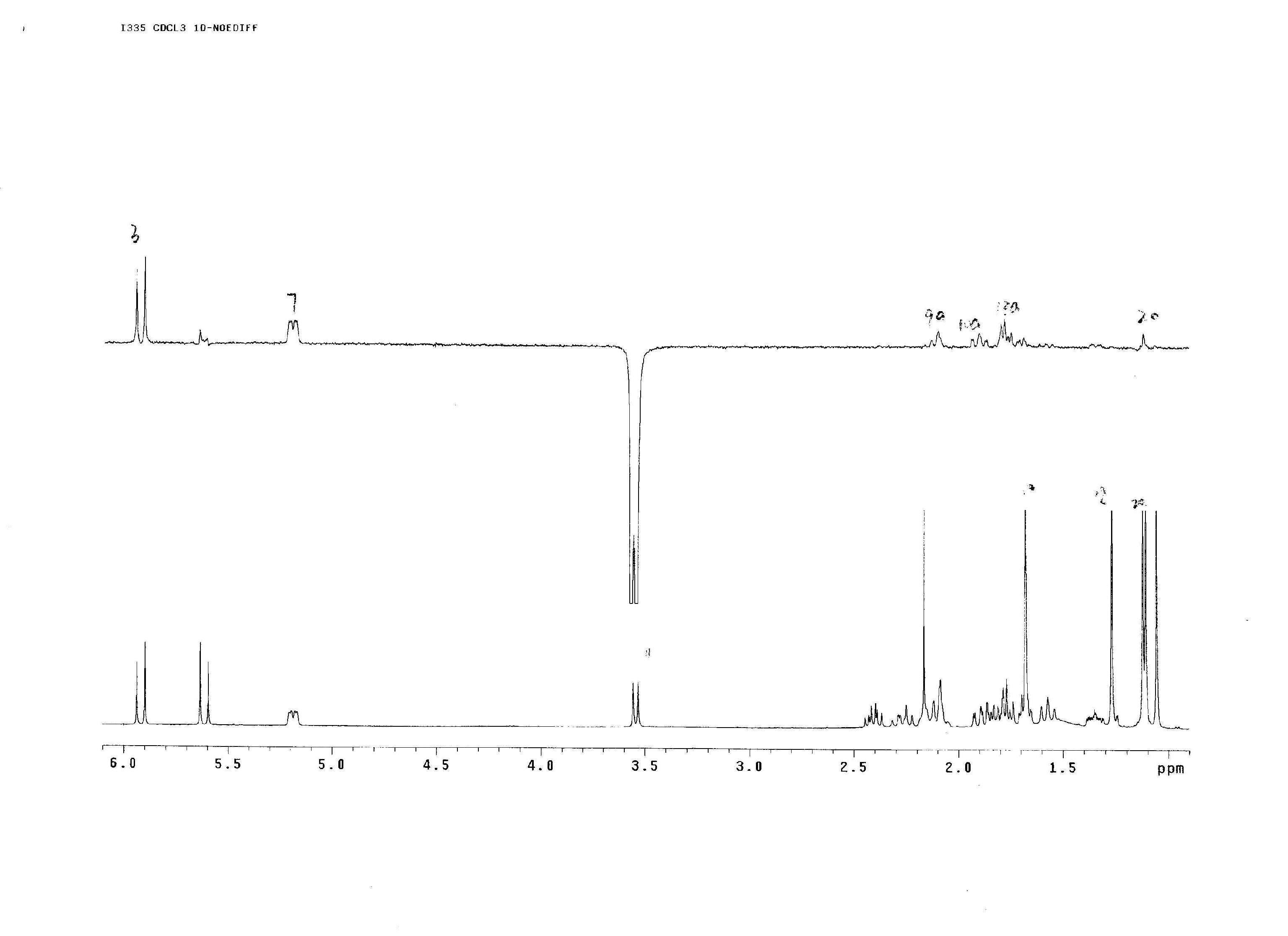


**Figure S7**. HRESIMS spectrum of sarcophytrol M (**1**)


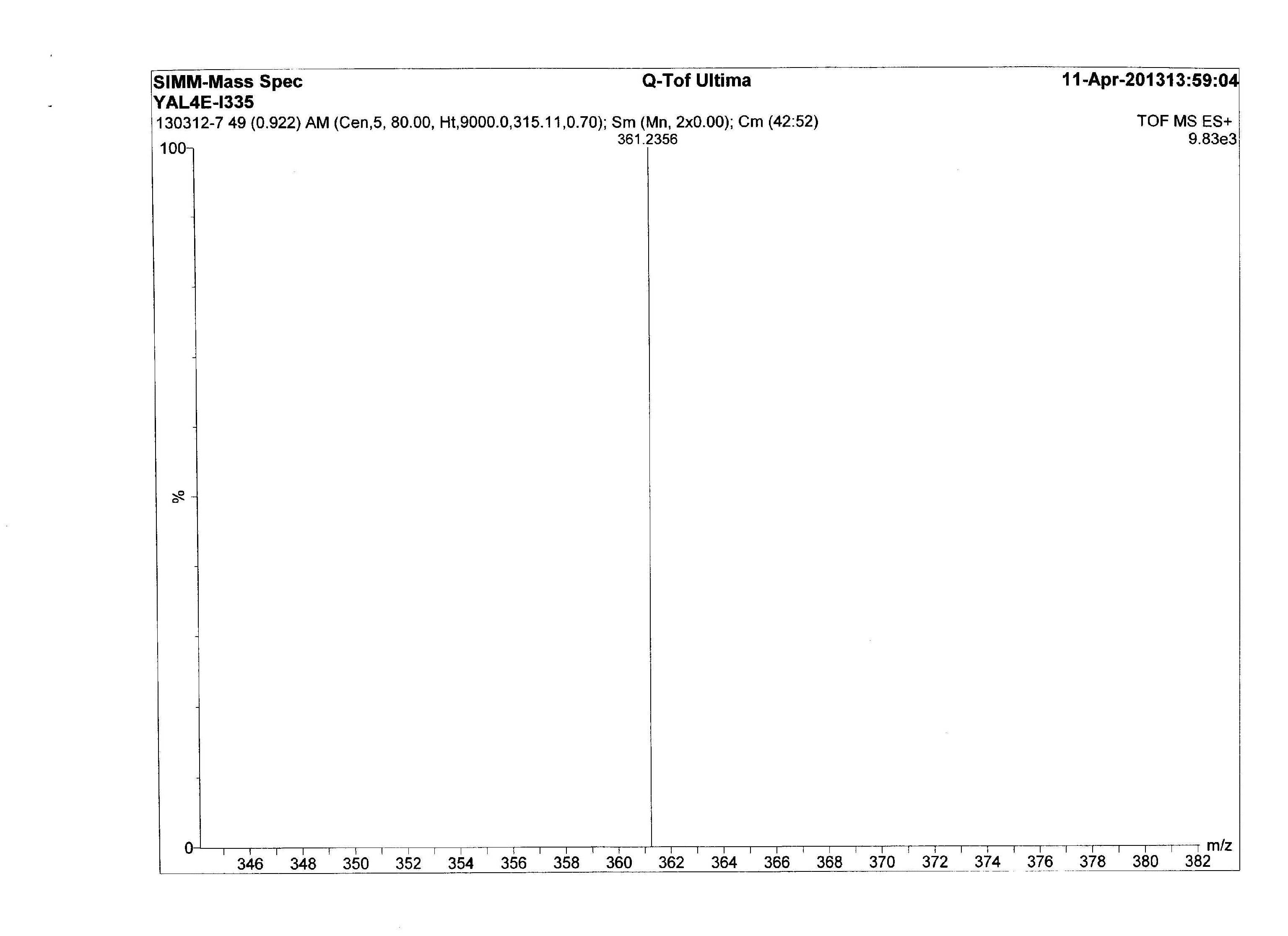


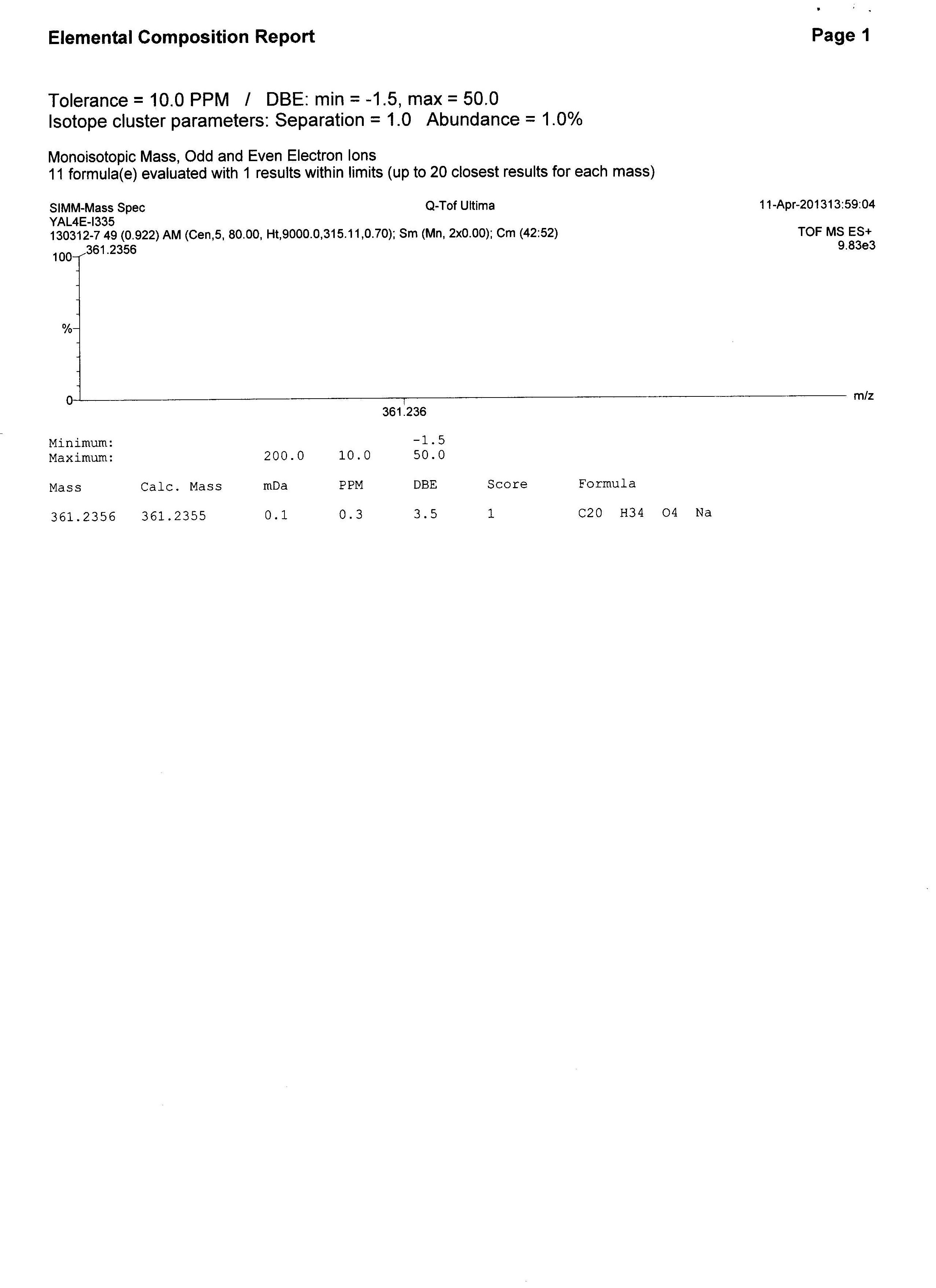


**Figure S8**. ^1^H NMR spectrum (500 MHz, CDCl_3_) of sarcophytrol N (**2**)

**Figure S9**. ^13^C NMR spectrum (125 MHz, CDCl_3_) of sarcophytrol N (**2**)

**Figure S10**. HMQC spectrum (500 MHz, CDCl_3_) of sarcophytrol N (**2**)

**Figure S11**. HMBC spectrum (500 MHz, CDCl_3_) of sarcophytrol N (**2**)

**Figure S12**. HRESIMS spectrum of sarcophytrol N (**2**)


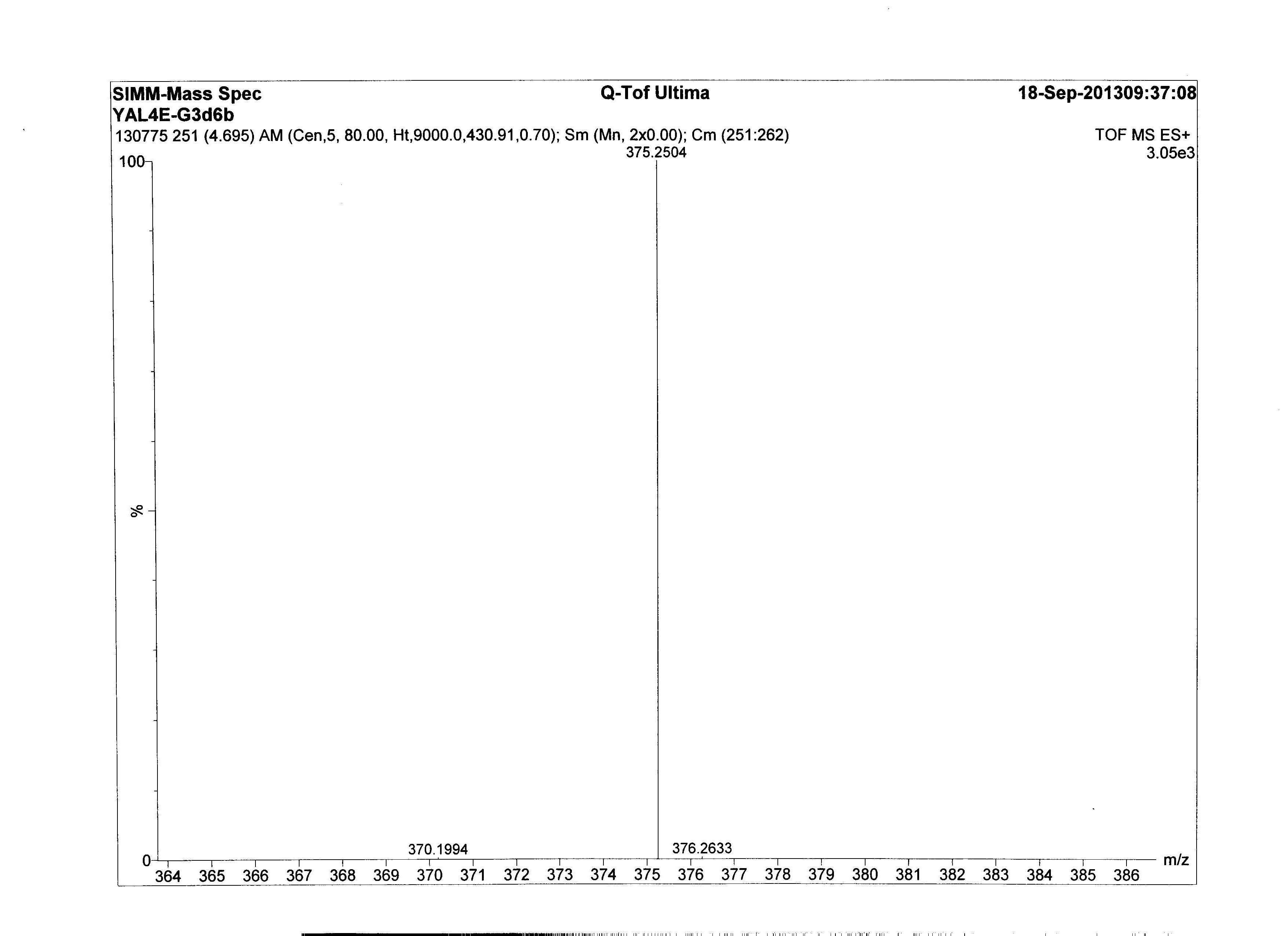


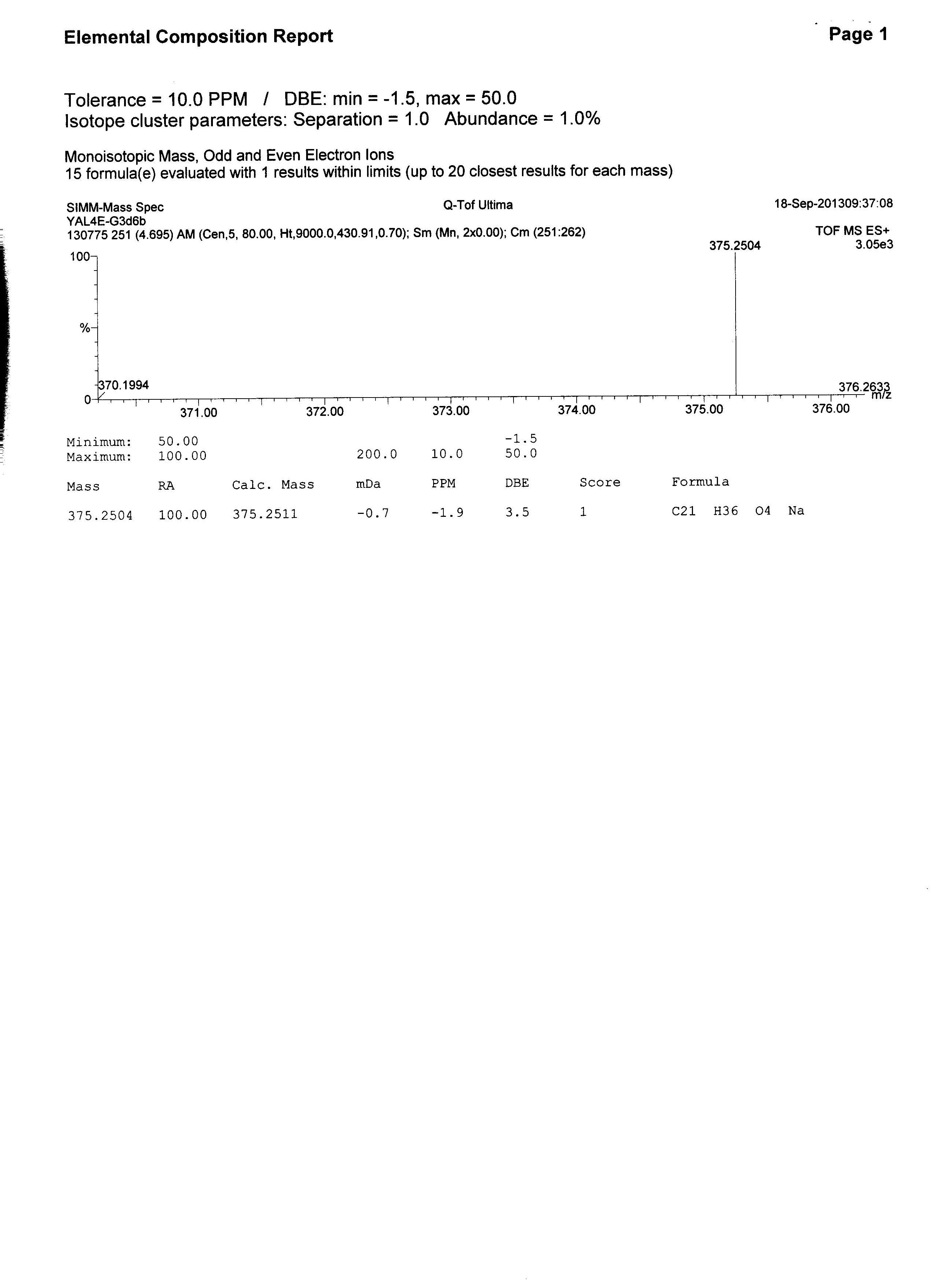


**Figure S13**. ^1^H NMR spectrum (500 MHz, CDCl_3_) of sarcophytrol O (**3**)

**Figure S8**. ^13^C NMR spectrum (125 MHz, CDCl_3_) of sarcophytrol O (**3**)

**Figure S15**. HMQC spectrum (500 MHz, CDCl_3_) of sarcophytrol O (**3**)

**Figure S16**. HMBC spectrum (500 MHz, CDCl_3_) of sarcophytrol O (**3**)

**Figure S17**. HRESIMS spectrum of sarcophytrol O (**3**)

**Figure S18**. ^1^H NMR spectrum (500 MHz, CDCl_3_) of sarcophytrol P (**4**)

**Figure S19**. ^13^C NMR spectrum (125 MHz, CDCl_3_) of sarcophytrol P (**4**)

**Figure S20**. HMQC spectrum (500 MHz, CDCl_3_) of sarcophytrol P (**4**)

**Figure S21**. NOE spectrum (400 MHz, CDCl_3_) of sarcophytrol P (**4**)

**Figure S22**. HRESIMS spectrum of sarcophytrol P (**4**)

**Figure S23**. ^1^H NMR spectrum (500 MHz, CDCl_3_) of sarcophytrol Q (**5**)

**Figure S24**. ^13^C NMR spectrum (125 MHz, CDCl_3_) of sarcophytrol Q (**5**)

**Figure S25**. HMQC spectrum (500 MHz, CDCl_3_) of sarcophytrol Q (**5**)

**Figure S26**. HMBC spectrum (500 MHz, CDCl_3_) of sarcophytrol Q (**5**)

**Figure S27**. ^1^H-^1^H COSY spectrum (500 MHz, CDCl_3_) of sarcophytrol Q (**5**)

**Figure S28**. NOE spectrum (400 MHz, CDCl_3_) of sarcophytrol Q (**5**)


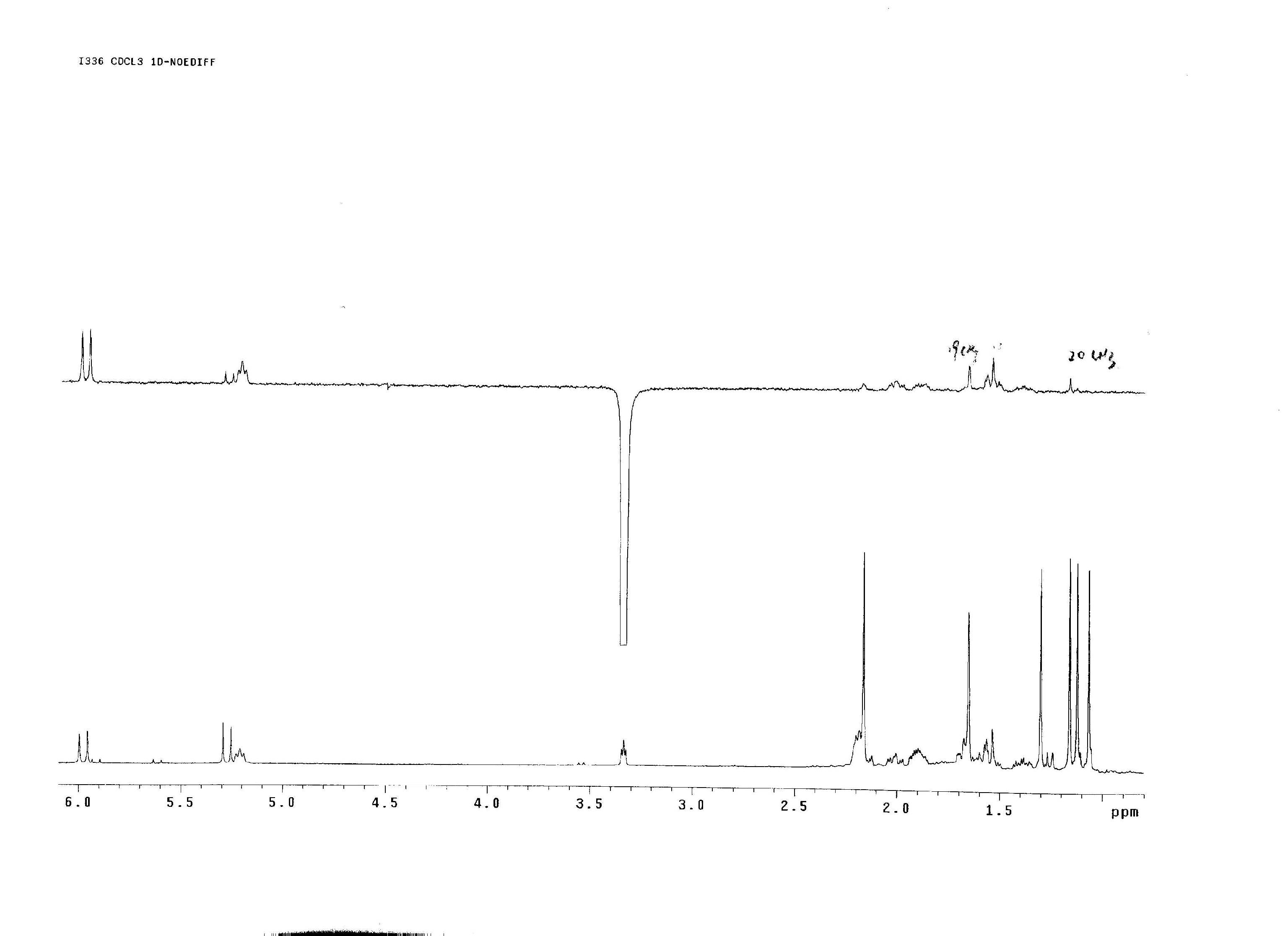


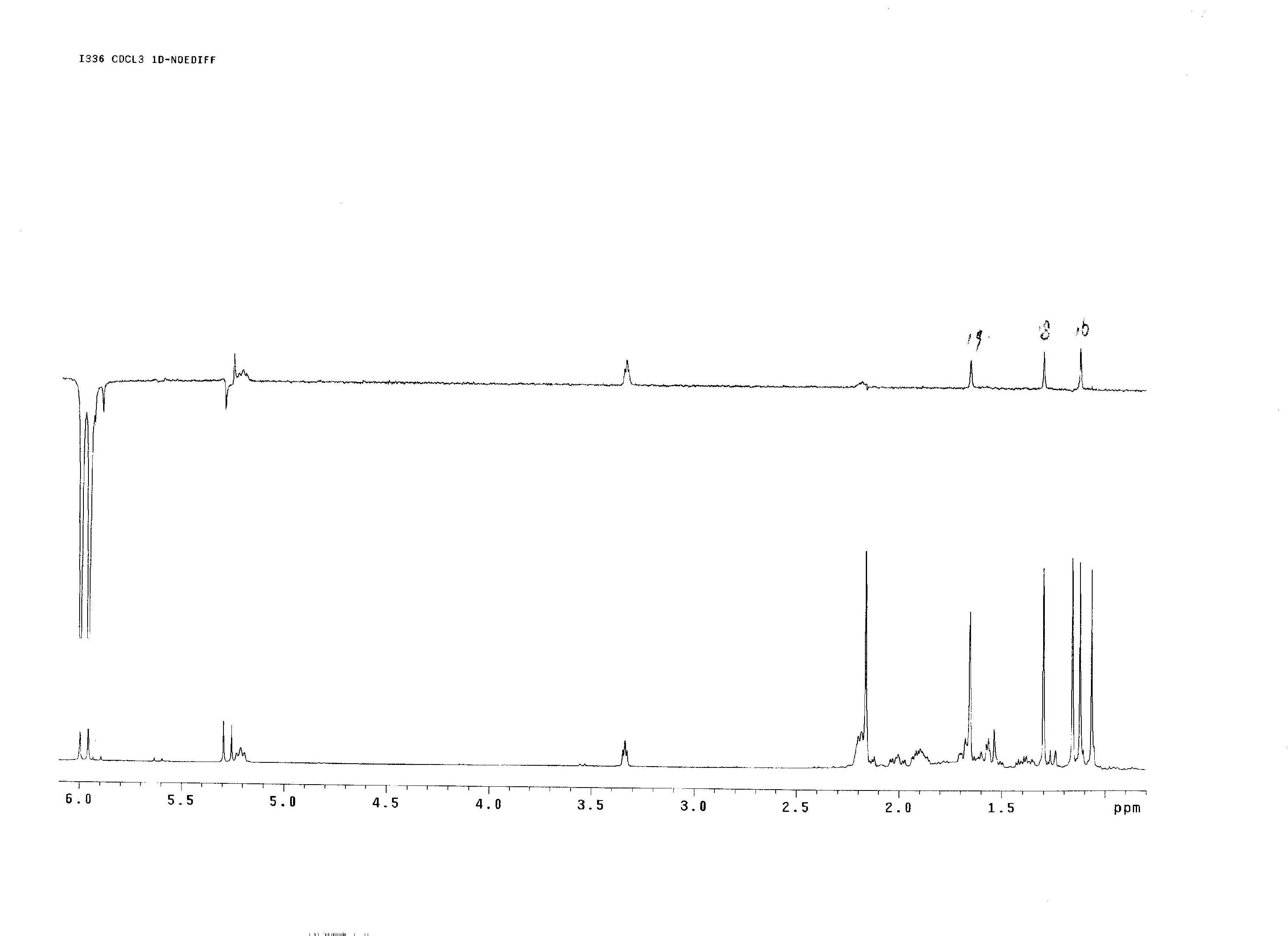


**Figure S29**. HRESIMS spectrum of sarcophytrol Q (**5**)


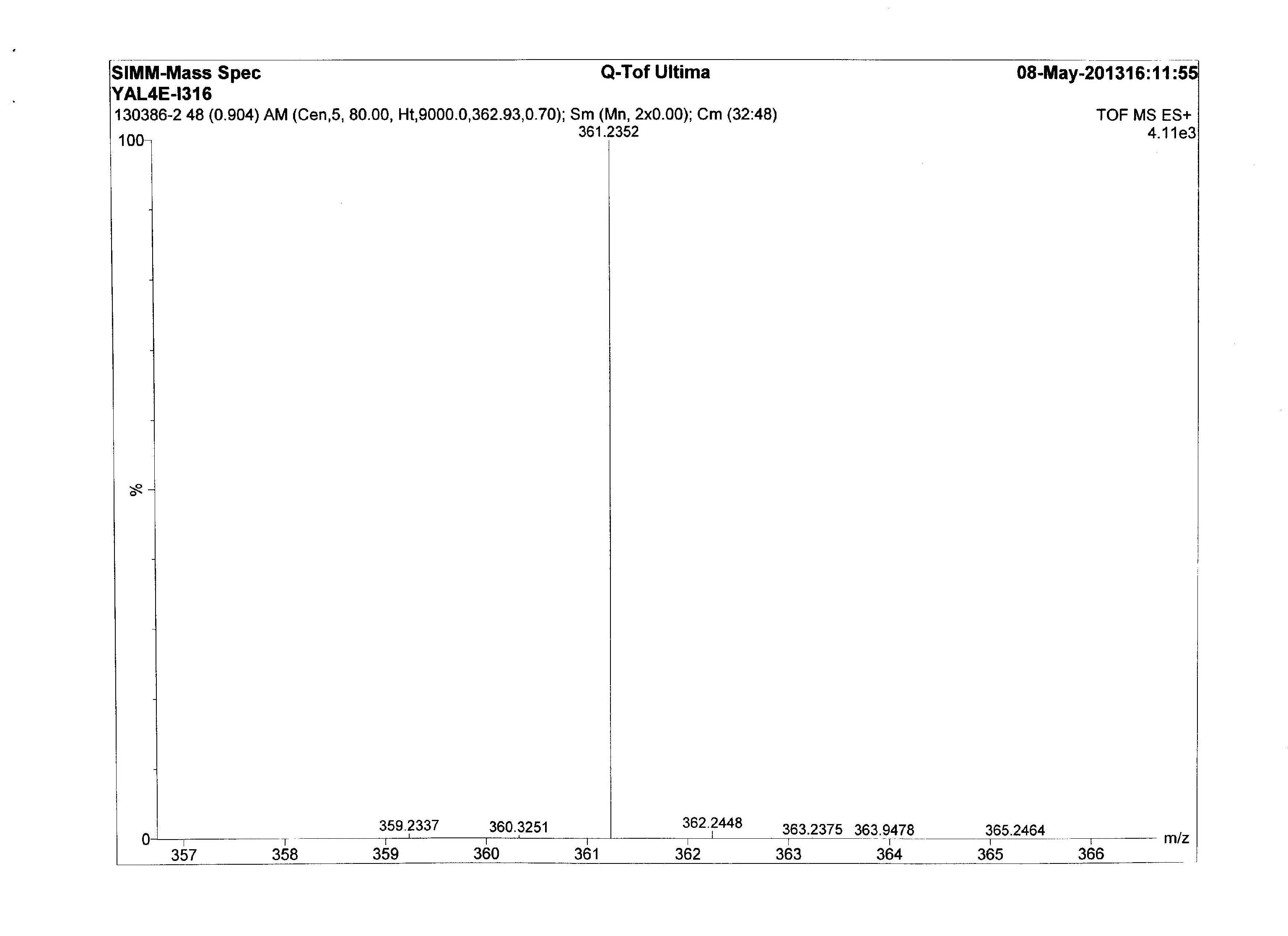


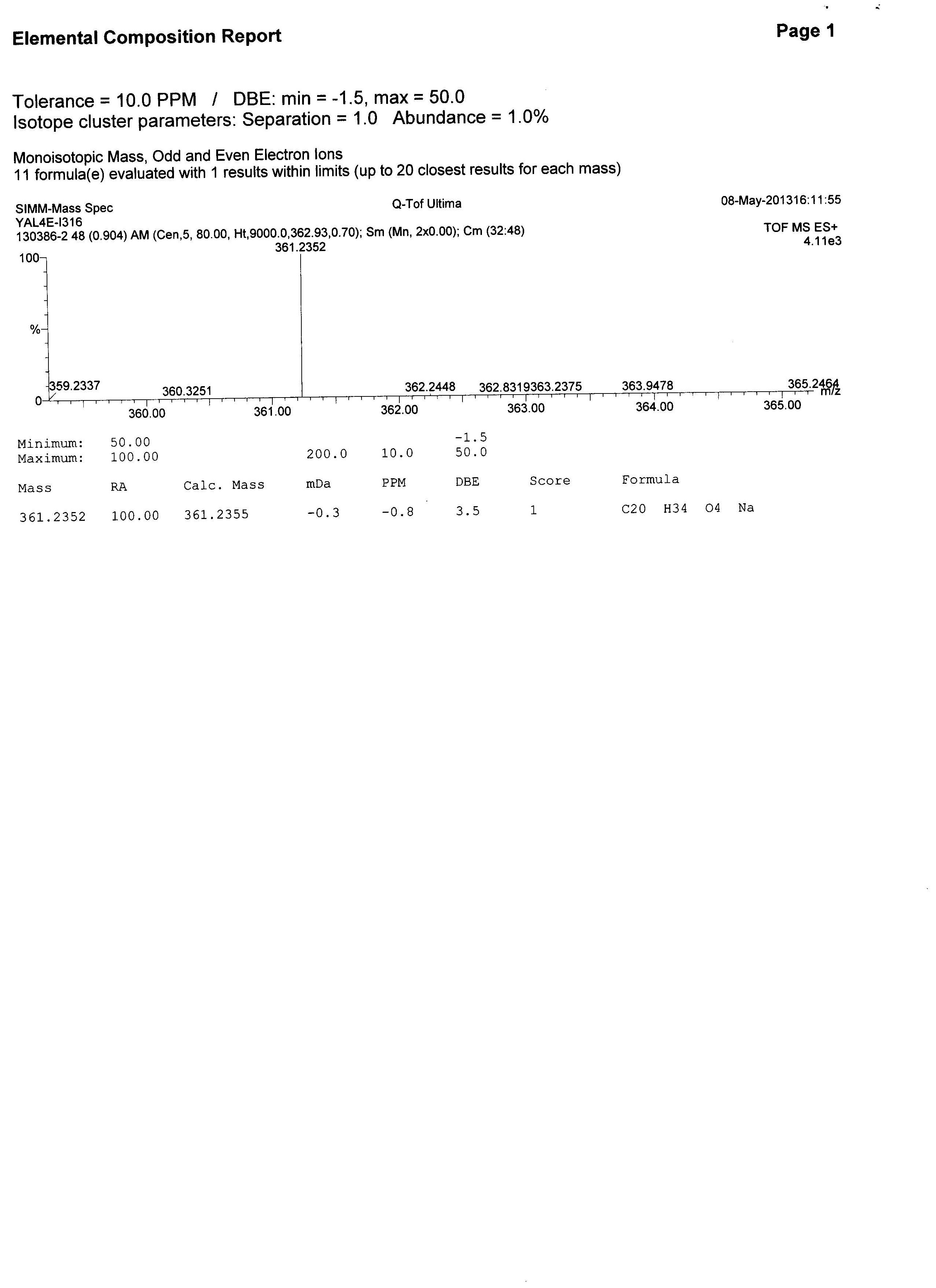


**Figure S30**. ^1^H NMR spectrum (500 MHz, CDCl_3_) of sarcophytrol R (**6**)

**Figure S31**. ^13^C NMR spectrum (125 MHz, CDCl_3_) of sarcophytrol R (**6**)

**Figure S32**. HMQC spectrum (500 MHz, CDCl_3_) of sarcophytrol R (**6**)

**Figure S33**. HMBC spectrum (500 MHz, CDCl_3_) of sarcophytrol R (**6**)

**Figure S34**. ^1^H-^1^H COSY spectrum (500 MHz, CDCl_3_) of sarcophytrol R (**6**)

**Figure S35**. ROESY spectrum (500 MHz, CDCl_3_) of sarcophytrol R (**6**)

**Figure S36**. HRESIMS spectrum of sarcophytrol R (**6**)


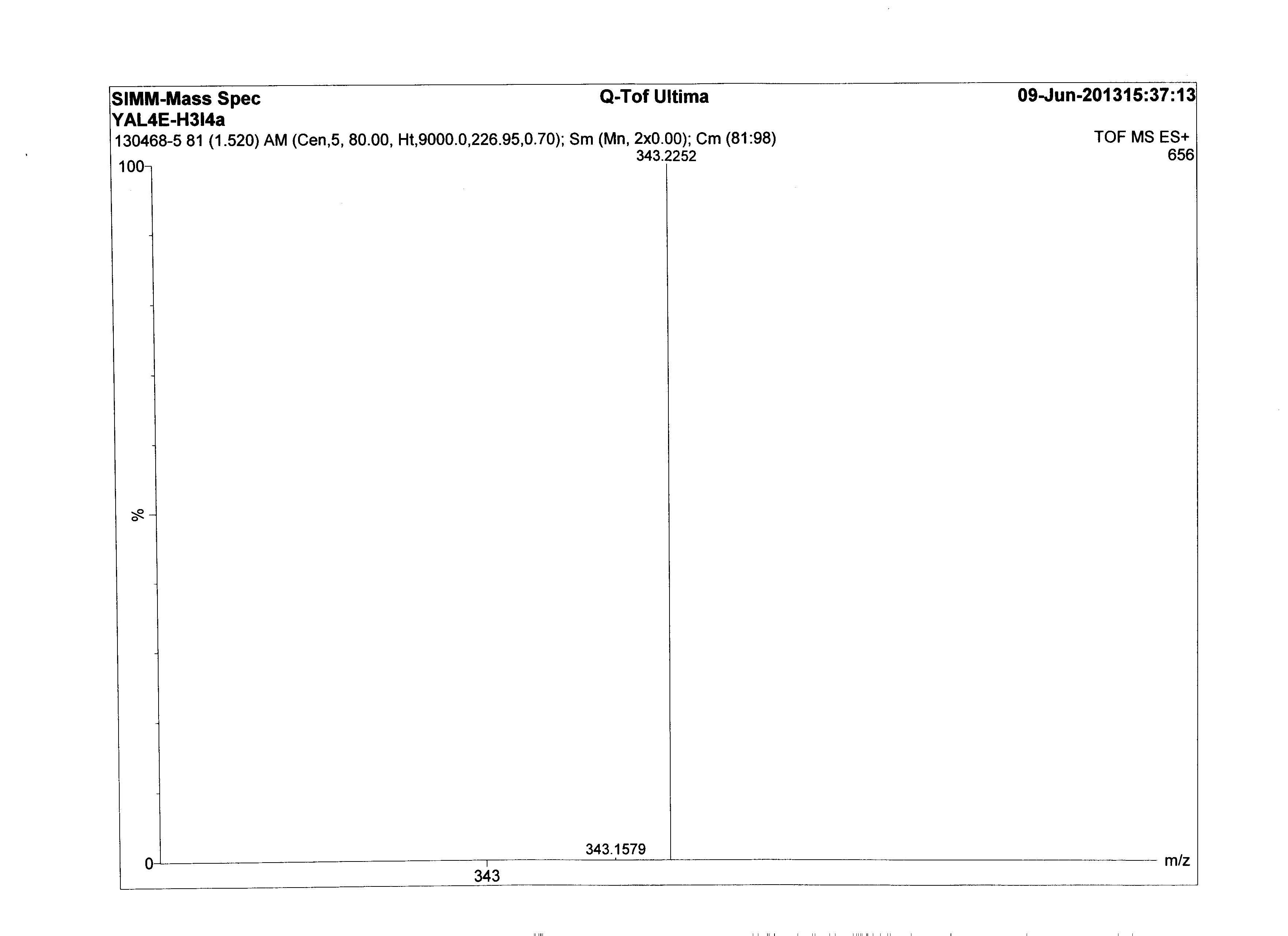


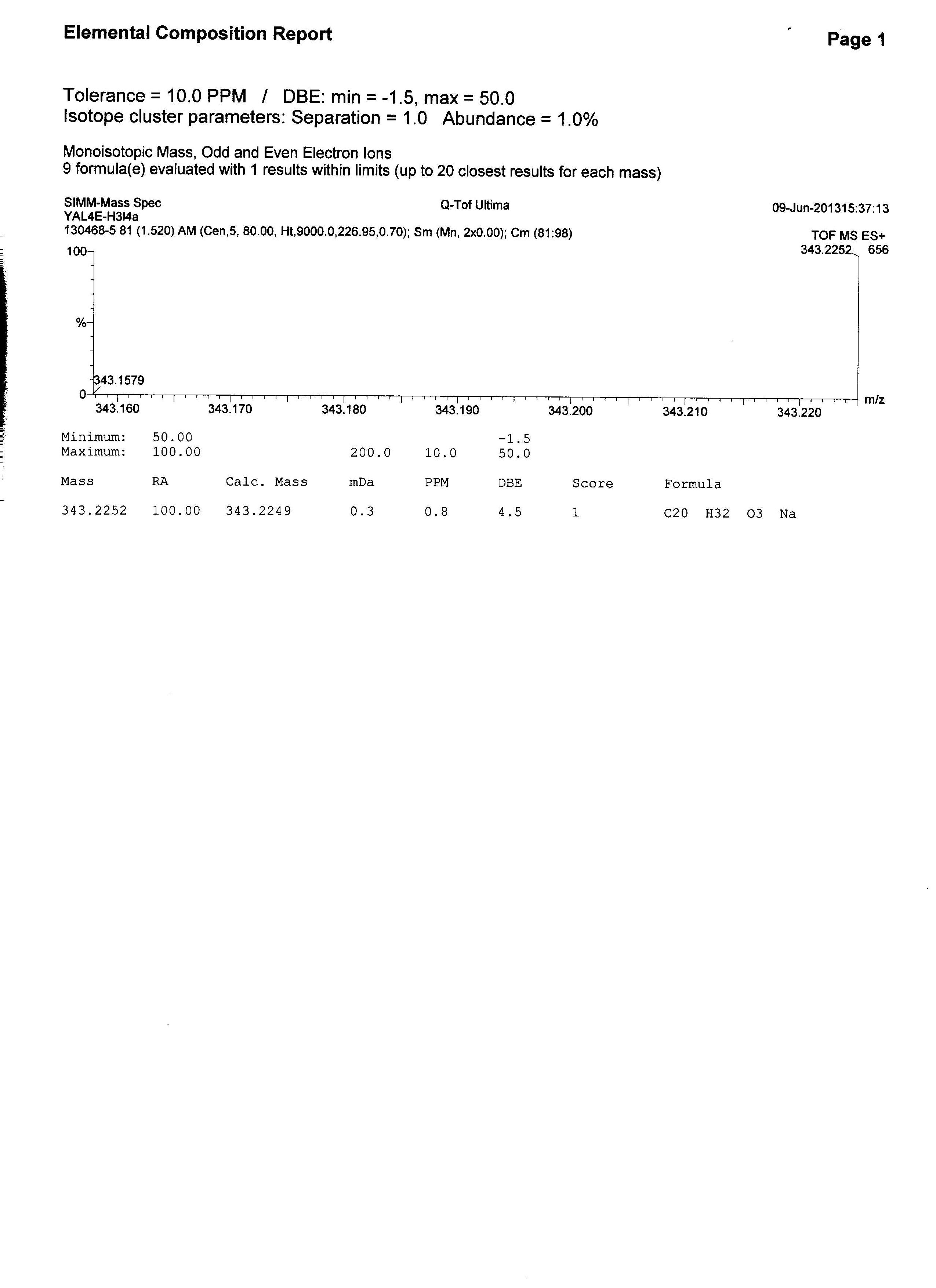


**Figure S37**. ^1^H NMR spectrum (500 MHz, CDCl_3_) of sarcophytrol S (**7**)

**Figure S38**. ^13^C NMR spectrum (125 MHz, CDCl_3_) of sarcophytrol S (**7**)

**Figure S39**. HMQC spectrum (500 MHz, CDCl_3_) of sarcophytrol S (**7**)

**Figure S40**. HMBC spectrum (500 MHz, CDCl_3_) of sarcophytrol S (**7**)

**Figure S41**. ^1^H-^1^H COSY spectrum (500 MHz, CDCl_3_) of sarcophytrol S (**7**)

**Figure S42**. ROESY spectrum (500 MHz, CDCl_3_) of sarcophytrol S (**7**)

**Figure S43**. ^1^H NMR spectrum (500 MHz, DMSO) of sarcophytrol S (**7**)

**Figure S44**. ^13^C NMR spectrum (125 MHz, DMSO) of sarcophytrol S (**7**)

**Figure S45**. HMQC spectrum (500 MHz, DMSO) of sarcophytrol S (**7**)

**Figure S46**. HMBC spectrum (500 MHz, DMSO) of sarcophytrol S (**7**)

**Figure S47**. ^1^H-^1^H COSY spectrum (500 MHz, DMSO) of sarcophytrol S (**7**)

**Figure S48**. NOE spectrum (400 MHz, DMSO) of sarcophytrol S (**7**)

**Figure S49**. HRESIMS spectrum of sarcophytrol S (**7**)


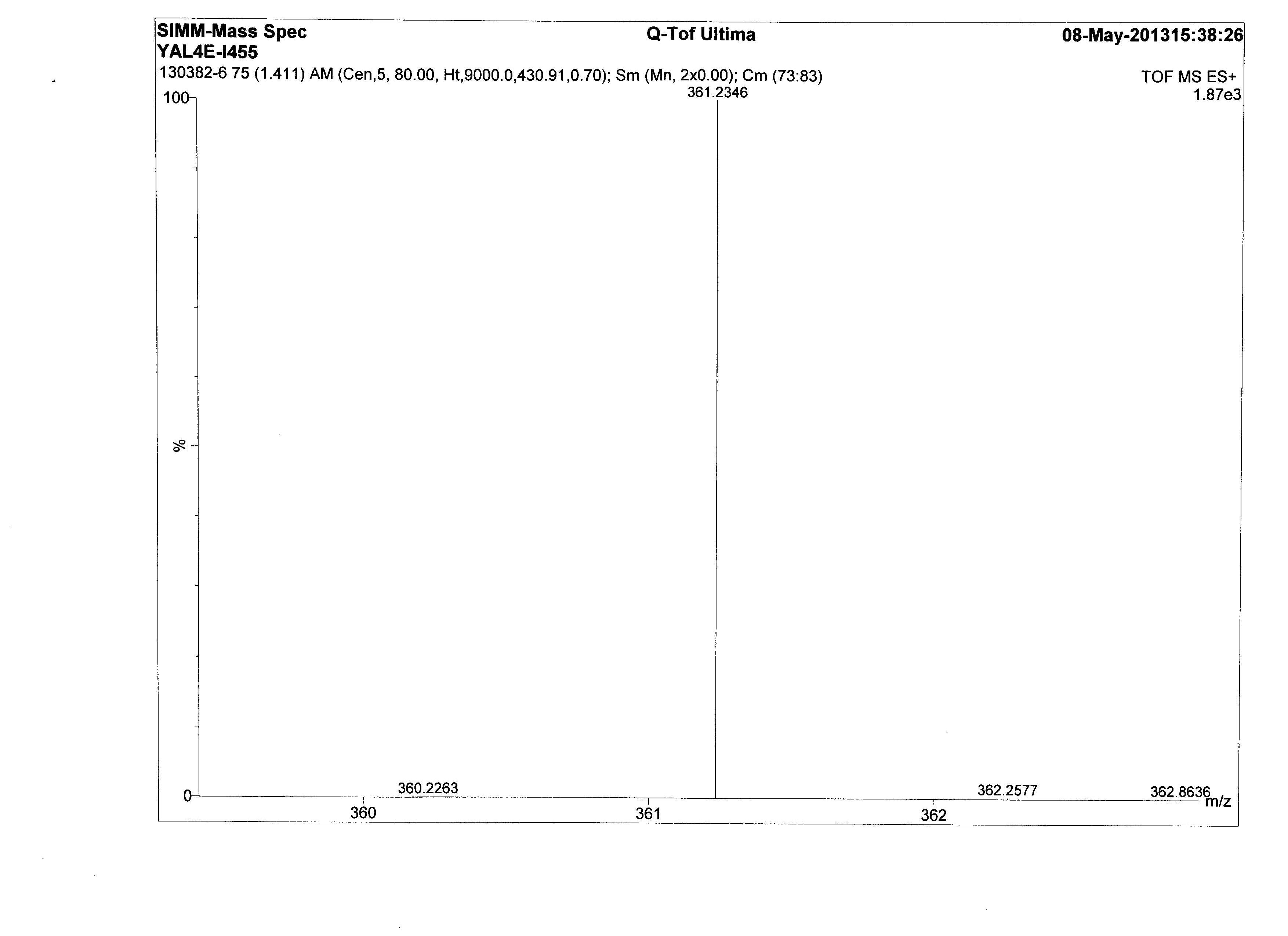


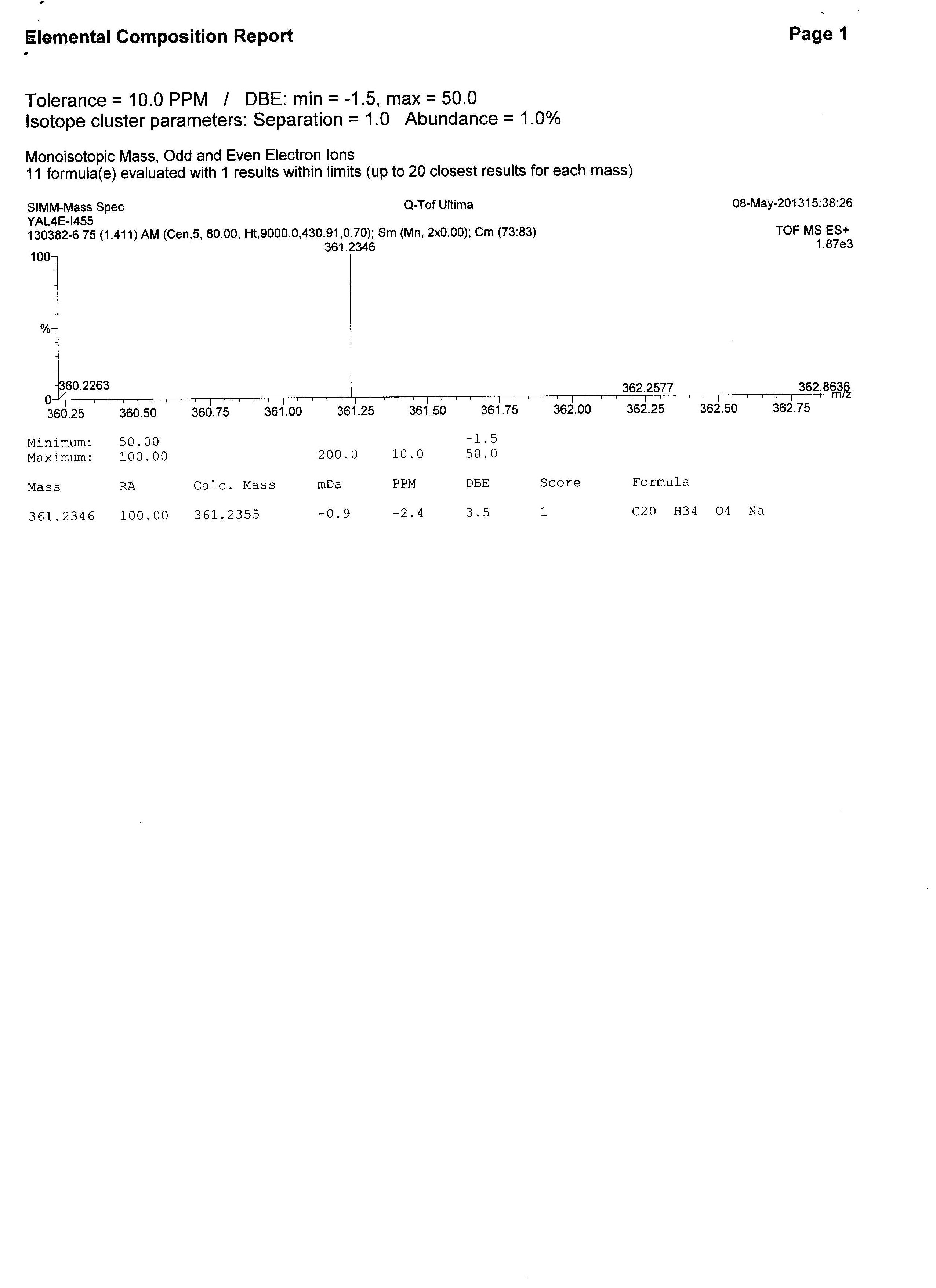


**Figure S50**. ^1^H NMR spectrum (500 MHz, CDCl_3_) of sarcophytrol T (**8**)

**Figure S51**. ^13^C NMR spectrum (125 MHz, CDCl_3_) of sarcophytrol T (**8**)

**Figure S52**. HMQC spectrum (500 MHz, CDCl_3_) of sarcophytrol T (**8**)

**Figure S53**. HMBC spectrum (500 MHz, CDCl_3_) of sarcophytrol T (**8**)

**Figure S54**. ^1^H-^1^H COSY spectrum (500 MHz, CDCl_3_) of sarcophytrol T (**8**)

**Figure S55** HRESIMS spectrum of sarcophytrol T (**8**)


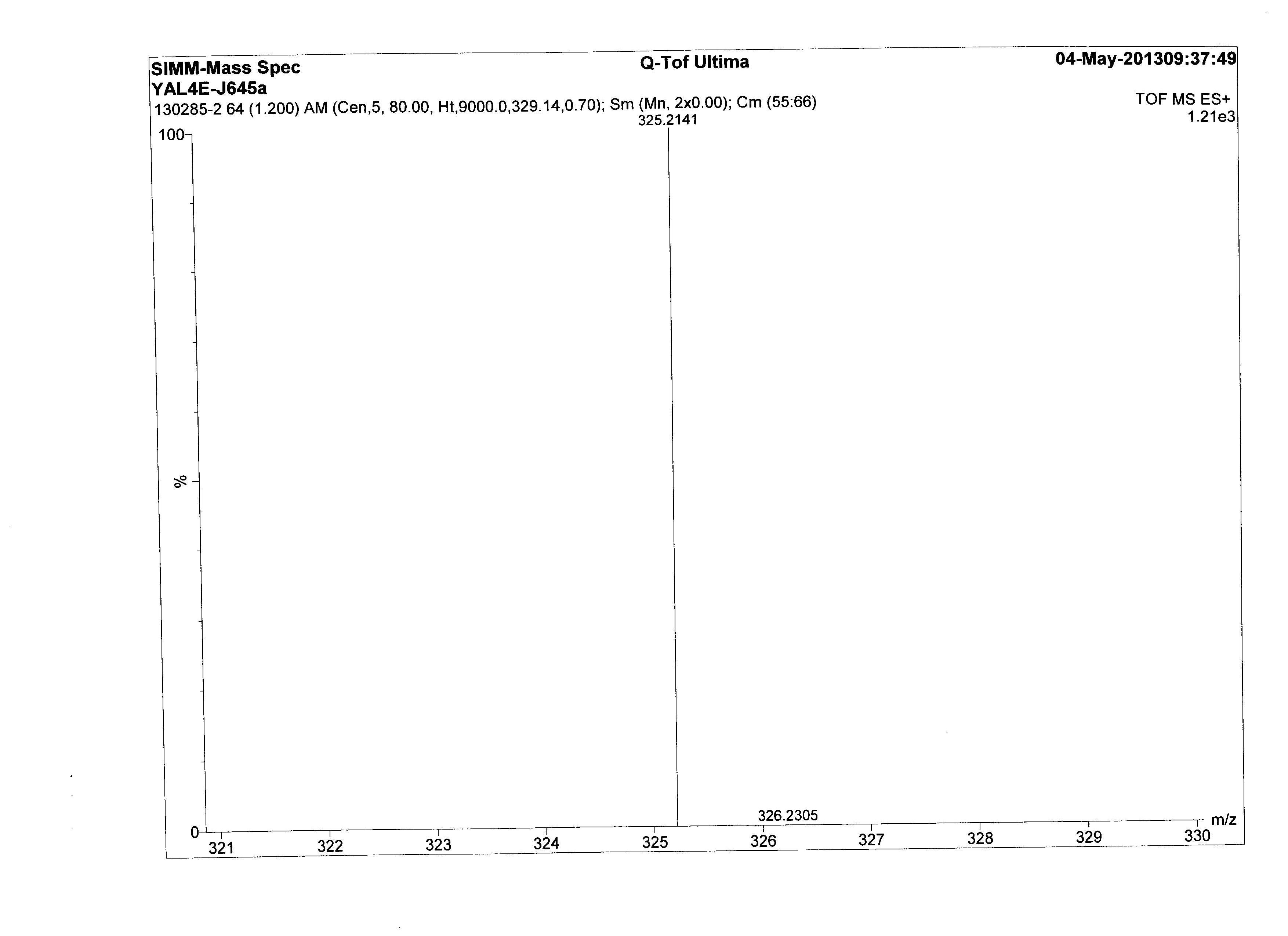


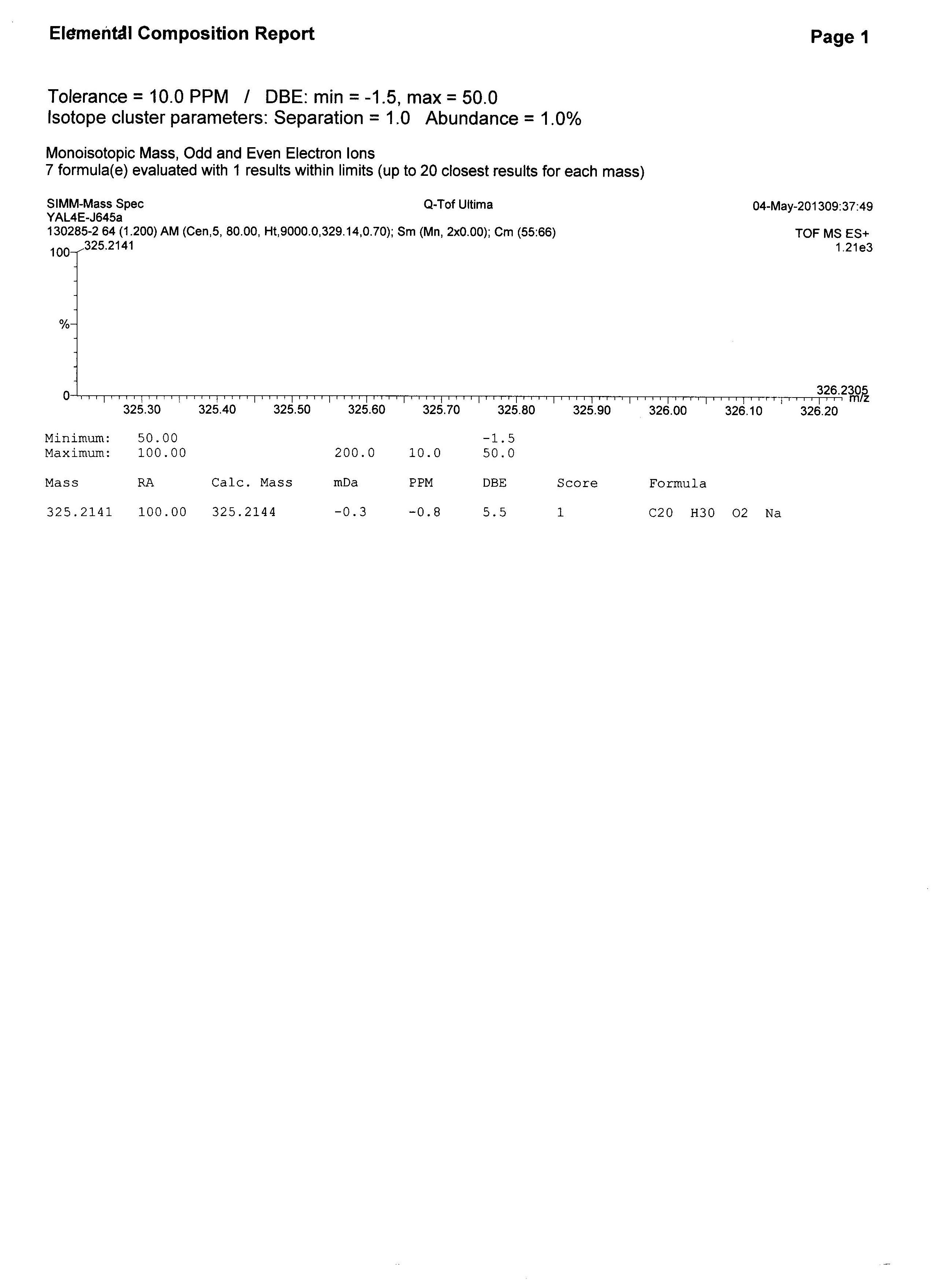


**Figure S56**. ^1^H NMR spectrum (500 MHz, CDCl_3_) of sarcophytrol U (**9**)

**Figure S57**. ^13^C NMR spectrum (125 MHz, CDCl_3_) of sarcophytrol U (**9**)

**Figure S58**. HMQC spectrum (500 MHz, CDCl_3_) of sarcophytrol U (**9**)

**Figure S59**. HMBC spectrum (500 MHz, CDCl_3_) of sarcophytrol U (**9**)

**Figure S60**. ^1^H-^1^H COSY spectrum (500 MHz, CDCl_3_) of sarcophytrol U (**9**)

**Figure S61**. ROESY spectrum (500 MHz, CDCl_3_) of sarcophytrol U (**9**)

**Figure S62**. HRESIMS spectrum of sarcophytrol U (**9**)


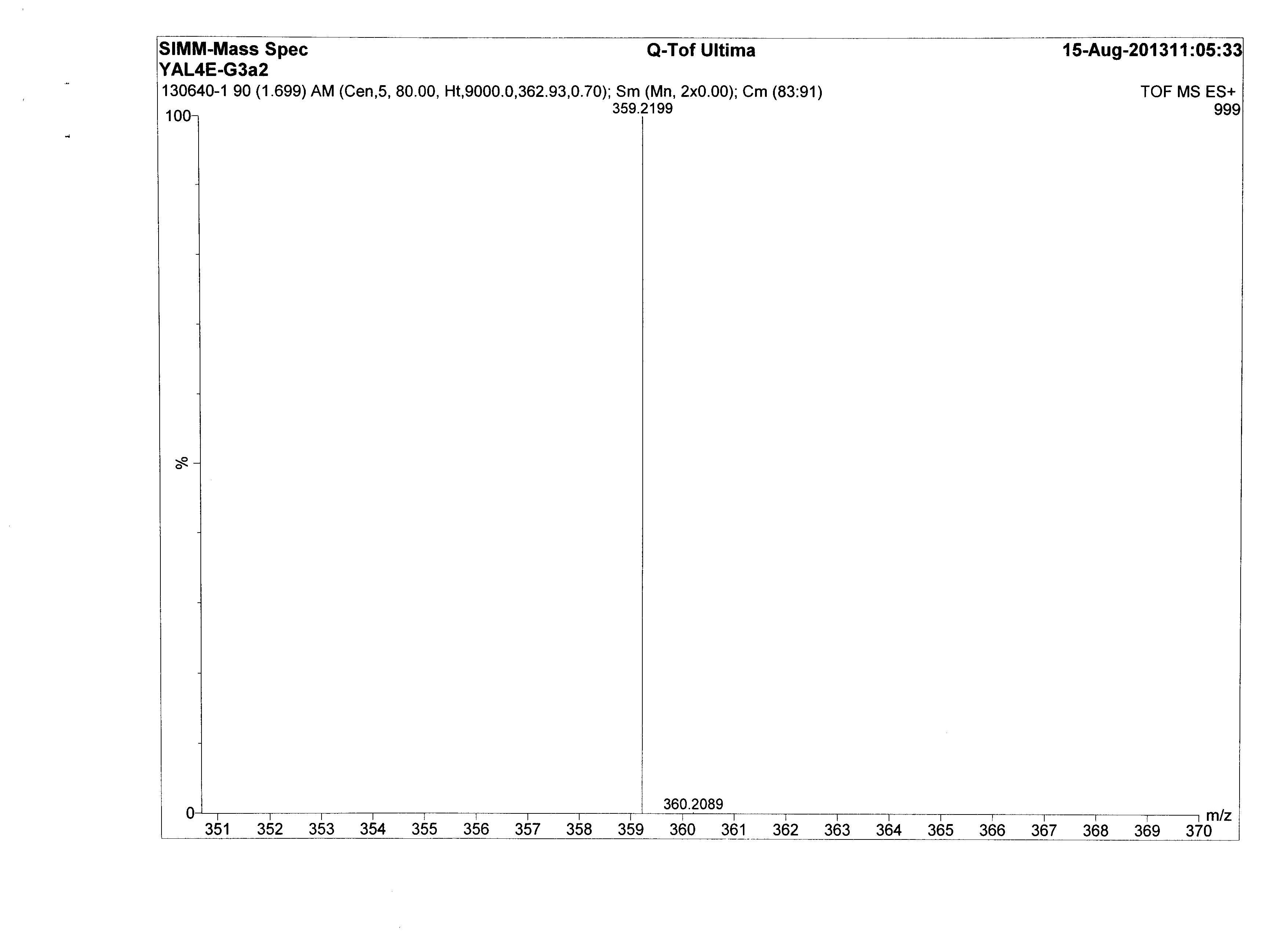


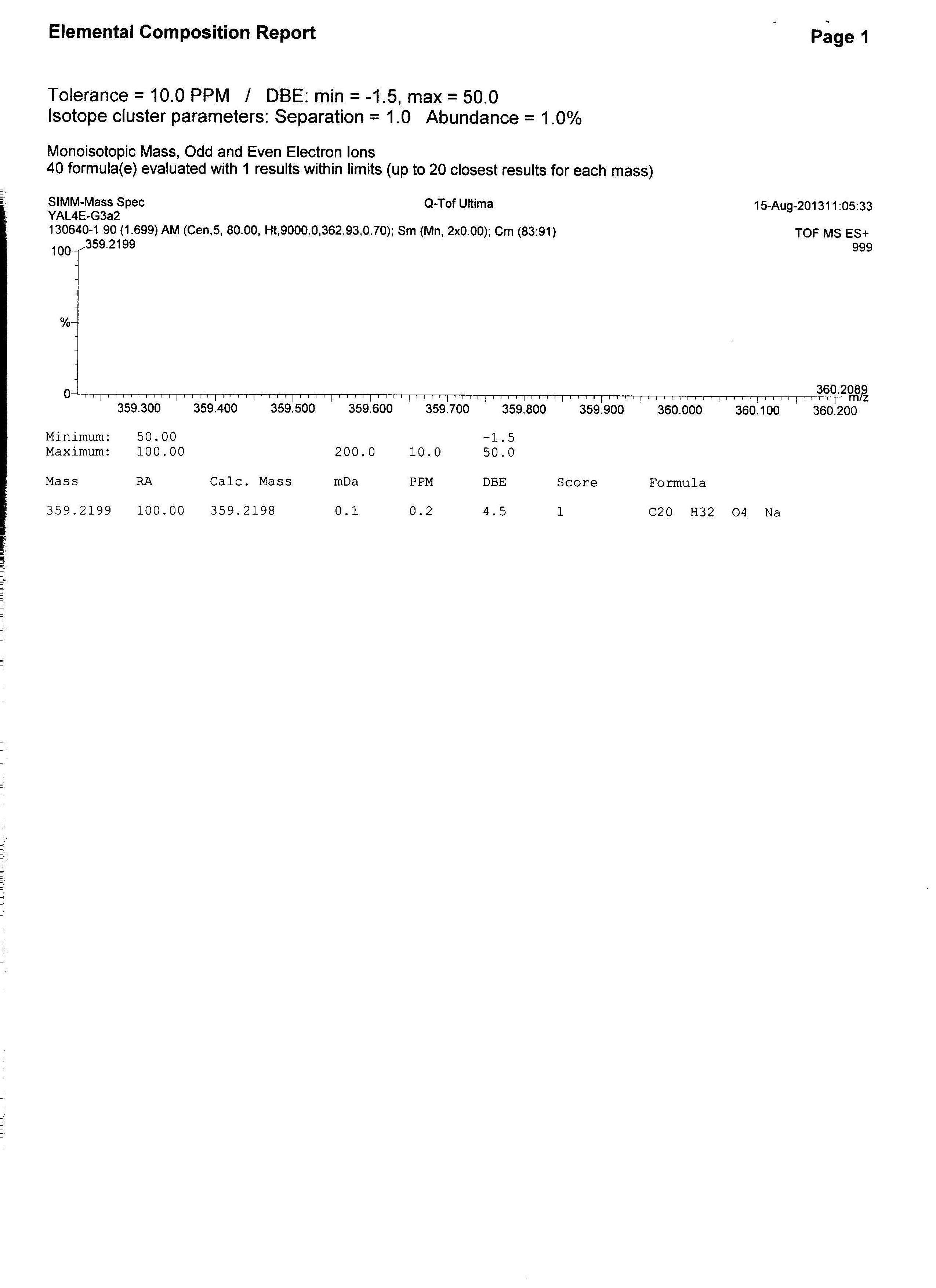


1. ^1^State Key Laboratory of Drug Research, Shanghai Institute of Materia Medica, Chinese Academy of Sciences, Shanghai 201203, China

   ^2^College of Material Science and Engineering, Central South University of Forestry and Technology, Changsha 410004, China

   ^#^These authors contributed equally to this work.

   ^*^Correspondence and requests for materials should be addressed to Y.-W. G. (email: ywguo@simm.ac.cn). [↑](#footnote-ref-2)
